# Atrial Cardiomyopathy: Pathophysiology, Diagnostic Approaches, and Prognostic Implications—A Narrative Review

**DOI:** 10.3390/jcm15166317

**Published:** 2026-08-15

**Authors:** Greta Barauskiene, Mindaugas Barauskas, Sandrita Simonyte, Jolanta Justina Vaskelyte

**Affiliations:** 1 Clinical Cardiology Laboratory, Institute of Cardiology, Lithuanian University of Health Sciences, Sukileliu av. 15, LT-50162 Kaunas, Lithuania; jolanta.vaskelyte@lsmu.lt; 2 Medical Academy, Lithuanian University of Health Sciences, A. Mickeviciaus 9, LT-44307 Kaunas, Lithuania; mindaugas.barauskas@lsmu.lt; 3 Laboratory of Molecular Cardiology, Institute of Cardiology, Lithuanian University of Health Sciences, Sukileliu av. 15, LT-50162 Kaunas, Lithuania; sandrita.simonyte@lsmu.lt

**Keywords:** atrium cardiomyopathies, morphometry and function aspects, electrophysiology aspects, diagnostics algorithms, biomarkers, genetic factors, prognosis

## Abstract

Atrial cardiomyopathy (ACM) is defined as any complex of structural, architectural, functional, electrophysiological, and molecular changes affecting the atria that may result in clinically significant health consequences. ACM can be caused by a variety of factors, including age-related changes, valvular or vascular disease, genetic diseases, congestive heart failure, metabolic diseases, cardiovascular disease (CVD) risk factors such as arterial hypertension (AH) or obesity, obstructive sleep apnea, and other infectious or noninfectious diseases predisposing to chronic inflammation. The diagnosis of ACM relies on several modalities, including electrocardiography, echocardiography, cardiac magnetic resonance imaging (MRI), computed tomography (CT), electroanatomical mapping (EAM), genetic studies, and biomarkers, which can detect and characterize structural, mechanical, and electrical atrial dysfunction. These changes often include structural atrial remodeling (fibrosis), abnormal structure of the atrial wall and its components, and contractile and electrical dysfunctions. When assessing aspects of ACM, structural changes in the atria such as left atrium (LA) size and fibrosis; LA architectural changes such as the expression of remodeling; changes in LA mechanics such as echocardiographic stress indices; changes in reservoir function and changes in contraction; biological factors determining changes in biomarkers; possible genetic predispositions and higher expression of certain genes encoding certain proteins; and arrhythmogenic factors associated with a higher risk of atrial fibrillation (AF) and stroke and a worse short- and long-term prognosis are very important. When considering the challenges of diagnosing ACM, it should be noted that without standardized diagnostics, most ACM diagnostic situations remain primarily research tools rather than practical clinical diagnostic methods. This review critically evaluates the evidence and translational gaps in the diagnosis of ACM, synthesizing the emerging role of advanced diagnostics and their clinical and prognostic implications as a key future tool for individual risk stratification.

## 1. Introduction

Atrial cardiomyopathy (ACM) has emerged as an important concept in cardiovascular medicine because of its strong association with AF, ischemic stroke, HF, and systemic thromboembolism. It refers to structural, architectural, contractile, and electrophysiological abnormalities affecting the atrial myocardium that may occur independently or in association with other CVDs. The growing proportion of older adults, AH, obesity, diabetes, and cardiovascular disorders such as aortic stenosis and mitral regurgitation have contributed to the growing clinical significance of ACM.

Traditionally, atrial abnormalities were mainly considered secondary consequences of AF. However, recent evidence suggests that ACM itself may precede and promote the development of atrial arrhythmias and thromboembolic complications. The pathological process involves atrial fibrosis, myocyte hypertrophy, inflammation, oxidative stress, endothelial dysfunction, and electrical remodeling, all of which contribute to impaired atrial conduction and mechanical dysfunction. These changes create a substrate that favors arrhythmogenesis and blood stasis, particularly within the left atrial appendage, thereby increasing the risk of stroke even in the absence of documented AF.

Advances in imaging modalities such as echocardiography and cardiac MRI, together with the identification of circulating biomarkers, have improved understanding of the structural and functional changes associated with ACM. Furthermore, the European Heart Rhythm Association (EHRA) has proposed a histopathological classification system that categorizes ACM according to the predominant underlying pathological changes, emphasizing the heterogeneous nature of the disease.

Despite growing recognition of ACM, important challenges remain regarding its definition, diagnosis, prognostic significance, and therapeutic management. A clearer understanding of its underlying mechanisms may facilitate earlier detection, improved risk stratification, and the development of targeted therapies aimed at preventing AF, stroke, and progressive cardiac dysfunction. Therefore, this review aims to summarize the current evidence regarding the pathophysiology, classification, clinical manifestations, diagnostic approaches, and prognostic implications of ACM.

## 2. Definition of ACM

ACM is defined as any complex of structural, architectural, functional, electrophysiological, and molecular changes affecting the atria that may result in clinically significant health consequences. These changes often include structural atrial remodeling (fibrosis), abnormalities of the atrial wall and its components, contractile dysfunction (myocardial contractility impairment, systolic dysfunction, or reduced ejection fraction), and electrical dysfunction (causing changes in the electrical signals that coordinate the heartbeat). ACM is often associated with cardiac complications such as AF but can also be a primary significant factor contributing to important health conditions such as stroke, heart failure (HF), or atrial arrhythmias [1].

## 3. Causes of ACM

ACM can be caused by a variety of factors, including age-related changes, valvular or vascular disease, genetic diseases, congestive heart failure, metabolic diseases, CVD risk factors such as AH or obesity, obstructive sleep apnea, and other infectious or noninfectious diseases predisposing to chronic inflammation. Sometimes, ACM can be difficult to distinguish from atrial changes caused by other heart diseases; however, there are isolated forms of ACM that are often associated with genetic factors. In clinical practice, it is important to recognize ACM because it is a driver of disease progression, posing an independent risk of AF or stroke. The presence of ACM may indicate a complex disease process that requires specific management strategies beyond the treatment of arrhythmia itself [1].

## 4. Pathophysiology of ACM

ACM is defined as structural, architectural, contractile, or electrophysiological alteration of the atria that can produce clinically significant manifestations such as AF, thromboembolism, stroke, and HF [2]. The pathophysiology of ACM involves a complex interaction between electrical, structural, metabolic, and inflammatory remodeling processes:

Structural remodeling: (1) Chronic pressure or volume overload (e.g., AH, valvular disease, heart failure) causes atrial stretch and enlargement. (2) Atrial myocytes undergo hypertrophy, degeneration, and apoptosis. (3) Fibroblast activation leads to excessive extracellular collagen deposition, resulting in atrial fibrosis, the hallmark of atrial cardiomyopathy. Fibrosis disrupts normal conduction pathways and impairs atrial mechanical function [3,4].

Electrical remodeling: (1) Alterations in ion channels shorten atrial action potential duration and refractory periods. (2) Abnormal calcium handling and gap-junction dysfunction promote conduction, heterogeneity and re-entry circuits. (3) These changes increase susceptibility to atrial arrhythmias, especially AF (“AF begets AF”) [3,5].

Inflammation and oxidative stress: (1) Systemic inflammation, oxidative stress, obesity, diabetes mellitus, and aging contribute to endothelial dysfunction and myocyte injury. (2) Cytokines and reactive oxygen species activate profibrotic signaling pathways, accelerating fibrosis and remodeling [3,4].

Mechanical dysfunction: (1) Fibrosis and myocyte dysfunction reduce atrial compliance and contractility. (2) Impaired atrial transport function leads to blood stasis, particularly in the left atrial appendage, increasing thrombus formation and embolic stroke risk [3,6].

Neurohormonal and metabolic mechanisms: (1) Activation of the renin–angiotensin–aldosterone system (RAAS), sympathetic nervous system, and transforming growth factor-β pathways promotes fibrotic remodeling. (2) Emerging evidence also implicates adipose tissue infiltration, amyloid deposition, and gut microbiota-related metabolites in disease progression [3,4].

Contemporary research has identified multiple interconnected molecular pathways that drive atrial fibrosis, inflammation, oxidative stress, metabolic dysfunction, and electrical remodeling.

Among all profibrotic signaling pathways, the TGF-β/SMAD pathway is considered the master regulator of atrial fibrosis [7]. Mechanical stretch, aging, AH, diabetes mellitus, and neurohormonal activation stimulate secretion of TGF-β1 from fibroblasts, macrophages, endothelial cells, and injured cardiomyocytes. Binding of TGF-β1 to TGF-β receptor II induces phosphorylation of TGF-β receptor I, activating intracellular SMAD2 and SMAD3 proteins. These phosphorylated SMADs complex with SMAD4 and translocate to the nucleus, where they regulate transcription of extracellular matrix (ECM) genes including collagen I, collagen III, fibronectin, connective tissue growth factor (CTGF), and periostin. Persistent activation promotes fibroblast-to-myofibroblast differentiation characterized by α-smooth muscle actin (α-SMA) expression, excessive ECM deposition, and atrial stiffening. Importantly, TGF-β signaling also suppresses matrix metalloproteinases while increasing tissue inhibitors of metalloproteinases (TIMPs), impairing ECM turnover and accelerating fibrosis. Several animal studies demonstrate that inhibition of TGF-β signaling attenuates atrial fibrosis and reduces AF susceptibility [8].

The RhoA/ROCK pathway links mechanical stress to cytoskeletal remodeling and fibrosis. Mechanical overload activates the small GTPase RhoA, which subsequently activates ROCK1 and ROCK2. These kinases regulate fibroblast proliferation, actin cytoskeleton organization, focal adhesion assembly, myofibroblast differentiation, and collagen synthesis. ROCK signaling also enhances TGF-β activity, NF-κB activation, inflammatory cytokine release, and oxidative stress. This creates a positive feedback loop amplifying fibrosis. ROCK additionally influences connexin expression, disrupting electrical coupling between atrial myocytes [9].

Mitochondrial dysfunction is an important component of ACM pathophysiology because the atria have exceptionally high energy demands owing to continuous excitation–contraction coupling. In ACM, mitochondrial dysfunction results from oxidative stress, calcium overload, impaired mitochondrial biogenesis, mitochondrial DNA damage, and defective mitophagy. These abnormalities reduce ATP production while increasing reactive oxygen species (ROS). Excess ROS activate multiple profibrotic pathways including TGF-β, MAPK, NF-κB, and NLRP3 inflammasome. Mitochondrial dysfunction also causes opening of the mitochondrial permeability transition pore, promoting apoptosis and replacement fibrosis. Recent work has demonstrated abnormalities in mitochondrial fusion/fission proteins MFN1, MFN2, OPA1, and DRP1. Disruption of mitochondrial dynamics further impairs atrial contractility. Studies of human atrial tissue have shown reduced respiratory chain complex activity, decreased PGC-1α expression, impaired fatty acid oxidation, and increased oxidative DNA damage. Collectively, these changes accelerate atrial remodeling [10].

MicroRNAs (miRNAs) are short non-coding RNAs that regulate post-transcriptional gene expression and have emerged as critical modulators of atrial remodeling. miR-21 is consistently upregulated in atrial fibrosis. Inhibition of miR-21 reduces fibrosis in experimental AF. The miR-29 family normally suppresses ECM production. Loss of miR-29 therefore accelerates atrial fibrosis. miR-133 normally inhibits connective tissue growth factor. Reduced miR-133 expression promotes fibroblast activation and fibrosis. miR-30 targets TGF-β signaling. Reduced expression enhances profibrotic gene transcription. miR-328 is associated primarily with electrical remodeling. Overexpression suppresses L-type calcium channel expression, shortening atrial refractory periods and increasing AF susceptibility. miR-1 regulates connexin-43 and Kir2.1 potassium channels, and altered expression contributes to conduction abnormalities. Collectively, dysregulated miRNAs integrate inflammatory, metabolic, and profibrotic signaling into persistent atrial remodeling [11].

The interaction between epicardial adipose tissue (EAT) and the atrial myocardium has attracted increasing attention because EAT has emerged as an active endocrine and paracrine organ rather than a passive fat depot. Unlike subcutaneous fat, EAT directly contacts the atrial myocardium without a fascial barrier, allowing diffusion of cytokines and adipokines. In obesity, diabetes, and metabolic syndrome, EAT becomes inflamed and secretes pro-inflammatory cytokines such as IL-1β, IL-6, TNF-α, and MCP-1; profibrotic mediators such as TGF-β, activin A, and CTGF; and adipokines such as leptin, resistin, and adiponectin (reduced). These mediators activate atrial fibroblasts through TGF-β/SMAD, NF-κB, JAK/STAT, MAPK, and RhoA/ROCK. Additionally, adipocyte infiltration into the atrial myocardium disrupts myocardial fiber continuity and creates conduction heterogeneity. Inflammatory macrophages within EAT release matrix metalloproteinases and ROS, further promoting fibrosis and electrical remodeling. Advanced imaging studies demonstrate strong correlations between increased EAT volume and atrial fibrosis on MRI, AF burden, recurrence after catheter ablation, and left atrial dysfunction. Emerging evidence also indicates that extracellular vesicles and exosomes released from EAT transport miRNAs and inflammatory mediators directly into atrial tissue, amplifying pathological remodeling [12].

The ACM process creates a vicious cycle in which atrial remodeling predisposes to AF, and AF further worsens remodeling and fibrosis [3,4]. The morpho-functional substrate that leads to ACM includes atrial enlargement, fibrosis with impaired systolic and diastolic properties of the atrial myocardium, abnormal electrophysiological features that promote the development of AF, which further worsens atrial remodeling, and atrial endothelial dysfunction [13,14,15,16,17]. In patients with HF, cardiac volume and pressure overload promotes atrial wall tension and cardiomyocyte stretch, aggravating atrial injury [18,19]. At the ultrastructural level, ACM can be characterized by cardiomyocyte abnormalities without sarcomeres, glycogen accumulation, chromatin disorders, mitochondrial dysfunction, overproduction of reactive oxygen species and impaired calcium processing, increased collagen deposition and fibroblast proliferation, infiltration of adipose tissue and inflammatory cells, deposition of glycosphingolipids and amyloid, alterations in excitation–contraction interactions and autonomic nervous system distribution [14].

Thus, the atrial myopathic process is caused and maintained by a combination of physiological and pathological phenomena, such as aging, inflammation, oxidative stress, fibrosis and atrial wall stretching. These factors are characterized by an increased neurohormonal element, including activation of the adreno–angiotensin–aldosterone system and mitogen-activated protein kinase pathway, increased transforming growth factor beta and matrix metalloproteinases, autonomic system remodeling, and secretion of adipokines that regulate the fibroblastic and inflammatory environment in epicardial adipose tissue, all of which contribute to the development of ACM [20,21,22].

## 5. Classification of ACM

A consensus document of the EHRA and other heart rhythm associations and organizations has proposed a classification system for ACM [23]. According to the EHRA guidelines, ACM is divided into four classes based on their histological characteristics and predominant pathological features (primary cardiomyocyte-dependent, primary fibroblast-dependent, mixed cardiomyocyte–fibroblast-dependent, and primary non-collagenous) [23]. Several issues limit the practical clinical application of this classification. First, because the system is histologically based, it is technically necessary to have atrial tissue samples to accurately assign individual patients to a specific class. Second, there is a significant overlap within each etiological class of ACM. Most ACMs can have features of multiple classes, with only one (amyloidosis) being classified into a single class, and 6 of 13 being classified into at least 3 of the 4 classes. Thus, for most types of ACM, it is not possible to unambiguously assign patients to a class without histopathological evaluation. There is also no clear consensus regarding this classification; even if a patient is unambiguously classified into one of the four classes, it is unclear what is to be done with this information [23].

The EHRAS classes represent the dominant pathological process rather than exclusive findings. Secondly, different regions of the same atrium may belong to different classes. Thirdly, patients may transition between classes as disease progresses [24].

EHRAS Class I is characterized by cardiomyocyte-dependent remodeling and predominantly affects atrial myocytes; fibrosis is minimal, and common findings include hypertrophy, vacuolization, myocytolysis, and altered contractile proteins. Electrical remodeling often precedes structural fibrosis.

EHRAS Class II is characterized by fibrosis-dependent remodeling and is dominated by collagen deposition; fibrosis separates myocyte bundles, producing slow conduction and conduction block, frequently associated with aging, hypertension, and pressure overload.

EHRAS Class III represents mixed cardiomyocyte/fibrotic remodeling, combining features of Classes I and II, and represents the most frequent phenotype in chronic AF and structural heart disease. This class produces both electrical instability and impaired mechanical function.

EHRAS Class IV is characterized by infiltrative remodeling with deposition of abnormal tissue rather than collagen, as seen in amyloidosis, inflammatory infiltrates (e.g., myocarditis), adipose infiltration, and storage diseases. Myocyte damage and fibrosis may coexist but are secondary to the infiltrative process.

The EHRA consensus emphasizes that ACM provides the pathological substrate linking cardiovascular disease with AF, atrial mechanical dysfunction, thromboembolism, and stroke [24].

The EHRAS classification was proposed primarily as a research and pathological framework rather than a clinical staging system. Because it requires histological assessment, routine clinical classification is currently limited, although modern imaging (especially cardiac MRI), electroanatomic voltage mapping, and circulating biomarkers may help identify corresponding phenotypes in the patients [24].

According to the latest HFA/ESC 2025 [25] recommendations, when assessing the phenomenon of ACM continuity, it should be noted that healthy atria are characterized by normal atrial structure and function, i.e., preserved reservoir, channel and amplifier-pump function, intact electrical conduction state and no significant fibrosis or atrial chamber enlargement. When the phenomenon of atrial remodeling occurs for some reason, an adaptive or maladaptive response to stressors occurs, i.e., electrical remodeling occurs (slow conduction in the atria, altered refractoriness), mechanical atrial dysfunction occurs, early fibrosis begins and as a result atrial enlargement develops. In the remodeling process, a subclinical stage may initially be detected, when clinical symptoms may not be present and this process may be potentially reversible. This remodeling process is often triggered by factors such as valvular disease, HF, AH, diabetes, obesity, sleep apnea, aging, and inflammatory processes. Later, ACM develops, which leads to electrical atrial dysfunction that can predispose to mechanical dysfunction, atrial enlargement, and atrial fibrosis. This document emphasizes that atrial fibrosis or AF alone is not sufficient. AF as a substrate is a multidomain disorder that includes electrophysiological changes, changes in atrial structure, changes in atrial mechanics, changes in biomarkers, and atrial tissue pathology. AF is defined as a manifestation of end-stage atrium failure involving a high degree of structural and atrial function impairment, loss of effective atrial contribution to cardiac output, increased atrial filling pressure, hemostasis phenomena, increased risk of thromboembolism, AF progression, and worsening HF outcomes. Atrial failure is conceptually analogous to ventricular failure, because of which the atria become unable to maintain normal hemodynamic and neurohormonal function. In assessing the etiology of primary and secondary atrial cardiomyopathy, the HFA/ESC 2025 [25] consensus agreed to distinguish two broad mechanistic categories. In primary ACM, intrinsic atrial disease such as genetic atrial myopathy, various ion channel disorders, isolated fibrotic atrial disease, and infiltrative or inflammatory atrial diseases predominate. In their course, atrial pathology occurs relatively independently of ventricular or systemic disease, and is characterized by disproportionate atrial dysfunction, early atrial fibrillation, and atrial fibrosis without significant ventricular pathology. In secondary ACM, atrial disease develops due to secondary causes, i.e., ventricular dysfunction, chamber pressure/volume overload, systemic disease, extracardiac factors such as HF with preserved ejection fraction, HF with non-preserved ejection fraction, mitral valve disease, aortic valve disease, AH, diabetes, obesity, chronic kidney disease, and sleep apnea. Secondary ACM can be caused by systemic and hemodynamic stress factors such as systemic inflammation, oxidative stress, neurohormonal activation, fibrosis, and atrial stretch/remodeling [25].

The HFA/ESC 2025 [25] document strongly emphasizes that most clinical atrial heart diseases are multifactorial and not purely primary or purely secondary in origin. This document (HFA/ESC 2025 [25]) also emphasizes the role of comorbidities and systemic factors. An important contribution of the 2025 consensus is the shift from an AF approach to a systemic disease model. The atrium is considered a target of chronic cardiometabolic and inflammatory stress. When assessing the main systemic factors that may predispose to the development of ACM, AH can be distinguished, during which chronic pressure overload is recorded, followed by atrial dilation, and even later its fibrosis phenomena appear and its elasticity is impaired. Obesity, as an expression of metabolic disease, contributes to the phenomena of inflammation of epicardial adipose tissue, the development of adipokine signaling disorders, oxidative stress, and increased atrial pressure/volume load. Diabetes, as a metabolic disease, contributes as a factor in glycemic disorders, during which microvascular dysfunction occurs, mitochondrial damage develops, and profibrotic signaling is impaired. In the presence of HF with preserved EF function (one of the strongest links), increased left ventricular filling pressure is determined, LA pressure overload occurs, stiffness develops and later progressive mechanical failure occurs. In the presence of sleep apnea, intermittent hypoxia develops, during which the sympathetic nervous system is activated, and pressure fluctuations and inflammation occur. In the presence of chronic kidney disease, the RAAS is activated, systemic inflammation develops, and fibrosis pathways appear. During aging, fibrotic tissue and fat infiltration develop in the atria, conduction heterogeneity occurs and atrial reserve decreases [25].

In assessing the pathophysiological framework, the HFA/ESC 2025 [25] consensus includes several interacting mechanisms involving hemodynamic stress phenomena characterized by increased or decreased pressure in the atria and their dilation, changes in cell signaling (RAAS activation, TGF-β pathways, oxidative stress, inflammatory phenomena), structural remodeling (fibrosis phenomena, atrial dilation, fatty infiltration), electrical atrial remodeling (slow conduction, heterogeneity, susceptibility to AF), mechanical atrial dysfunction (reduced reservoir/channel/contractile function), and clinical manifestations such as AF, stroke, HF, pulmonary hypertension, and atrial failure. The document emphasizes that AF is both a consequence of ACM and an acceleration of ACM, fibrosis and atrial dysfunction may precede the onset of AF, and sinus rhythm does not exclude clinically significant ACM [25].

## 6. Multimodal Evaluation

The modern concept of ACM has evolved substantially from a large pathophysiologic construct in 2016 into a clinically oriented, multimodal staging framework in the 2024 consensus update from the EHRA and collaborating societies. The transition reflects a shift from “what atrial disease is” toward “how atrial disease should be detected, staged, and clinically managed.” The 2016 EHRA/HRS/APHRS/SOLAECE consensus document established the foundational definition of ACM as: any complex of structural, architectural, contractile, or electrophysiological changes affecting the atria with the potential to produce clinically relevant manifestations [23].

The 2016 document emphasized several core principles, establishing that ACM is not synonymous with AF. AF may both result from and accelerate atrial remodeling.

Multiple systemic disorders contribute to atrial diseases such as AH, diabetes, obesity, heart failure, aging, sleep apnea, inflammatory and infiltrative disorders. Atrial disease involves several domains simultaneously such as fibrosis, electrical remodeling, contractile dysfunction, endothelial dysfunction, and thrombogenic remodeling.

The 2016 consensus also proposed the histopathologic EHRAS classification by histopathology: I—predominantly cardiomyocyte changes, II—predominantly fibrotic changes, III—combined cardiomyocyte pathology and fibrosis, IV—non-collagen infiltration (amyloid, fatty, inflammation substances). However, the EHRA system had limited direct clinical applicability because it relied heavily on tissue characterization rather than bedside phenotyping [23].

The 2024 EHRA clinical consensus (“ACM revisited evolution of a concept”) moved the field toward a multidimensional clinical staging model integrating biomarkers, atrial geometry, imaging, electrophysiology, arrhythmia burden, and clinical outcomes [1].

The major conceptual advance is that ACM is now viewed as a progressive disease continuum, like ventricular cardiomyopathy or heart failure staging. The 2024 document specifically states that: ACM may precede AF, AF is not required for diagnosis, and atrial disease itself may independently promote stroke, cognitive decline, heart failure, and mortality [1].

The 2024 EHRA proposal introduced a simplified ACM stage 1–3 model.

Stage 1—Early/Subclinical ACM with some characteristics of presence of risk factors or early atrial injury, minimal or absent structural remodeling; biomarker or electrical abnormalities may precede imaging changes. Typical findings include abnormal P-wave indices, elevated NT-proBNP, subtle LA strain reduction, mild atrial conduction slowing, with no sustained AF necessarily present. Clinical significance of this stage represents atrial vulnerability, and potentially reversible disease.

Stage 2—Manifest Structural/Electrical Remodeling with characteristics of clear evidence of atrial remodeling on imaging or electrophysiology. Findings may include LA enlargement, reduced atrial strain, fibrosis on LGE-MRI, low-voltage substrate mapping, impaired reservoir/conduit function, and interatrial conduction delay; AF is often present but not obligatory. Clinical implications consist of higher thromboembolic risk, increased AF recurrence after ablation, and progressive mechanical dysfunction.

Stage 3—Advanced ACM with characteristics of extensive structural and functional atrial disease with clinical complications. Features include persistent/long-standing AF, extensive fibrosis, severe atrial dysfunction, atrial standstill or marked electromechanical dissociation, stroke/systemic embolism, heart failure with preserved ejection fraction (HFpEF) association, and cognitive impairment association. Clinical significance includes advanced irreversible remodeling with major prognostic implications [1].

A major message of the EHRA consensus is that ACM should be evaluated using a multimodal approach, since each diagnostic modality interrogates different aspects of the disease. A clinical framework consistent with this EHRA consensus is needed. The EHRA consensus emphasizes that no single test establishes or excludes ACM. Instead, clinicians should integrate clinical risk factors, imaging findings, electrical characteristics, functional assessment, and select biomarker information to estimate the extent of atrial remodeling and guide decisions regarding rhythm control, catheter ablation, anticoagulation, and long-term surveillance [23].

These consensus documents consistently conclude that transthoracic echocardiography (TTE) is the first-line modality, cardiac MRI is preferred when detailed tissue characterization is clinically relevant, EAM is mainly reserved for patients undergoing AF ablation, circulating biomarkers are not yet ready for routine clinical diagnosis, and multimodal integration is the recommended approach because no single modality adequately captures the full spectrum of ACM.

## 7. Morphometric and Functioning Aspects of ACM

One of the morphometric indices of the atrium is the LA volume index (LAVI), a classical morphometric marker that measures the maximum LA volume indexed by body surface area (mL/m^2^). This index is widely used, easily determined using 2D echocardiography, but even more accurately measured using 3D echocardiography. LAVI predicts HF events and mortality and is a reliable marker of chronic atrial remodeling in various heart diseases. The clinical-practical limit (norm) of this index is ≤34 mL/m^2^, and higher LAVI values indicate mild/moderate/severe atrial damage. The maximum LAVI reflects cumulative LA remodeling, but early functional LA dysfunction may often be missed [26].

Another cardiac LA morphometric index is the LA minimum volume (LAVmin), which is expressed as a ratio (LAVmin/LAVImin), and shows a more significant hemodynamic correlation. It measures the smallest LA volume (usually at the end of diastole), indexed by body surface area.

One of the functional indicators of left atrial (LA) performance is LA strain, particularly peak atrial longitudinal strain (PALS), which is most assessed using two-dimensional speckle-tracking echocardiography, although three-dimensional echocardiography may also be used [27,28,29]. LA strain analysis evaluates atrial function and can detect early atrial dysfunction associated with left ventricular diastolic dysfunction (LVDD) [30]. It reflects the phasic deformation of the LA, including the reservoir phase (represented by PALS), the conduit phase, and the contractile (booster pump) phase.

Another indicator of LA function is LA sphericity/shape indices, which also have prognostic information. This index measures how spherical the LA has become (the ratio of measured LA volume to the volume of the sphere based on the maximal LA diameter). LA sphericity increases as atrial remodeling progresses. LA sphericity has been proposed as a potentially earlier and more sensitive indicator of AF-induced atrial remodeling compared to traditional markers such as LA enlargement [31].

Studies using 3D echocardiography have shown more accurate cardiac morphometry, phase volumes, and changes in atrial deformation. 3D echocardiography has been shown to more accurately measure LA volumes (max/min/pre-A), left ventricular (LV) ejection fraction, and 3D-derived deformation (on newer platforms). 3D echocardiography improves accuracy compared to 2D echocardiography, reduces geometric assumptions, and allows reliable measurement of LA minimal volume (LAVmin), which is important in research or serial observations [32,33,34].

## 8. Diagnostic Possibilities of ACM

Various methods, including electrocardiography (ECG), echocardiography, cardiac MRI, heart computed tomography (CT), electroanatomical mapping, genetic studies, and biomarkers, can detect and quantify structural, mechanical, and electrical atrial dysfunction [1]. Due to its heterogeneity, the clinical diagnosis of ACM is a challenging task. To date, there are no standardized recommendations for diagnosing suspected ACM. However, standardization could help to more accurately classify ACM and determine treatment directions to improve individualized patient management.

The 2016 diagnostic framework was primarily mechanistic and descriptive. Diagnostic emphasis was placed on histology/pathology, electrophysiologic abnormalities, structural remodeling, and AF association. Typical investigations include: ECG, echocardiography, electroanatomic mapping, histopathology, and MRI (emerging role). The limitation was lack of standardized clinical staging, prognostic thresholds, and integrated multimodal algorithms [35].

In 2024, the proposal shifts toward a layered clinical workflow [1].

Step 1—Identification of risk substrate by assessing AH, obesity, diabetes, sleep apnea, heart failure, aging, inflammatory disease, and genetic predisposition. This step recognizes ACM as a systemic disease phenotype.

Step 2—Electrical assessment with evaluation of ECG markers such as P-wave duration, P-wave terminal force in V1, PR interval, advanced interatrial block, and signal-averaged ECG abnormalities. Rhythm assessment: Holter monitoring, implantable loop monitoring, AF burden quantification, and electrical remodeling may precede anatomic remodeling.

Step 3—Structural/functional imaging with evaluation of echocardiography. Primary first-line tools: LA volume index, LA emptying fraction, and LA strain (reservoir strain particularly emphasized). Cardiac MRI is increasingly important for atrial fibrosis quantification, LGE-MRI, and atrial wall characterization. CT is useful for atrial geometry and epicardial adipose tissue assessment.

Step 4—Biomarker integration with evaluation biomarkers such as NT-proBNP, high-sensitivity troponin, inflammatory markers and fibrosis markers, and omics/genetic profiling. Biomarkers are intended to improve early detection, identify progression risk, and refine staging.

Step 5—Electrophysiologic characterization with evaluation of electroanatomic voltage mapping, conduction velocity assessment, fractionated electrogram analysis, and AF driver mapping to help define substrate severity.

Furthermore, uniform diagnostic criteria for ACM could be important for future studies. Combining and correlating multiple parameters seem to be useful in diagnosing ACM [36].

## 9. Diagnostic Algorithm

A key reason why clinical practitioners have an insufficient understanding of ACM is the lack of clear diagnostic criteria. According to the EHRA/HRS/APHRS/SOLAECE expert consensus, the classification of ACM relies on histopathological findings and the presence of fibrosis and/or non-collagen deposits [23]. This pathological diagnosis method is impractical for clinical applications. Here, we briefly introduce commonly used auxiliary inspection methods and feasible diagnostic criteria for assessing ACM. Effective assessment and characterization of ACM progression could enable a more informed and individualized strategy for addressing modifiable risk factors, as well as for the primary prevention of ACM complications.

**Figure 1:**
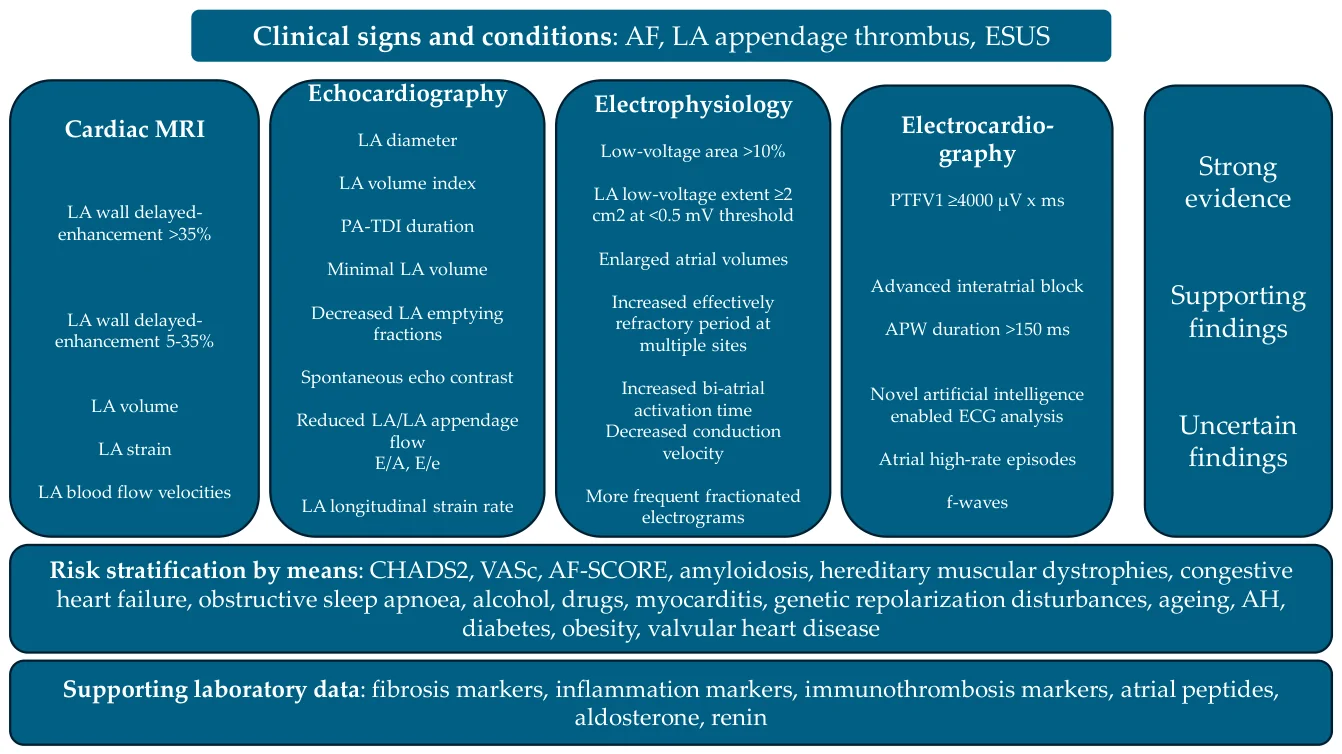
Algorithm for diagnosing atrial cardiomyopathy based on the results of previous studies. Adapted from Kreimer F et al., 2022 [36]. AF—atrial fibrillation; APW—amplified P-wave; ESUS—embolic stroke of undetermined source; LA—left atrial; PA—TDI-total atrial conduction time assessed by tissue Doppler imaging; PTFV1—P-wave terminal force in lead V1.

The main methods used to diagnose ACM are cardiac MRI, echocardiography, electrophysiological parameters, and electrocardiography. The following diagnostic algorithm for this pathology is most often used to diagnose ACM (Figure 1) [36].

Studies have shown that the pathogenesis of ACM is dominated by LA pressure overload, volume overload, fibrosis, electrophysiological changes, inflammatory reactions, and neurohormonal mechanisms [1].

The EHRA/HRS/APHRS/SOLAECE Expert Consensus on ACM emphasizes that no single diagnostic modality can fully characterize ACM. Instead, assessment should be tailored to the clinical question and integrate structural, functional, electrical, and biological information. The consensus does not recommend a universal diagnostic algorithm but outlines the complementary roles of imaging, electroanatomical assessment, and biomarkers [23].

## 10. Imaging Methods: 2D/3D Echo, MRI

Imaging techniques such as 2D/3D echocardiography and cardiac MRI are very commonly used to determine ACM [17,23]. Due to the widespread use of echocardiography, it has become the primary method for assessing ACM. Left atrial dilation is the most prominent feature in the remodeling process of ACM and the visual representation of LA diastolic dysfunction. The measurement of LA anteroposterior diameter through M-mode and subsequent two-dimensional echocardiography is simple and reproducible. Previous studies have used LA anteroposterior diameter as one of the morphological parameters for ACM [23]. However, due to the irregular three-dimensional structural characteristics of the atrium and the heterogeneity of atrial remodeling, measuring the atrial volume is more accurate for reflecting and assessing the true state of the atria than atrial diameter. In the case of LA enlargement, the increase in LA anteroposterior diameter is not directly proportional to the increase in the total LA volume, which becomes the main source of error in the assessment of LA volume by two-dimensional echocardiography [37]. Another study has shown that LA enlargement is associated with adverse clinical outcomes, independent of AF, such as stroke and heart failure [38]. However, in addition to intrinsic LA pathology, LA volume is also associated with a variety of factors, including left ventricular filling pressure, valvular heart disease, and so on; therefore, it is difficult to separate the impact of LA dilatation from left ventricular (diastolic) dysfunction [39]. Moreover, changes in LA volume are reversible; thus, LA volume does not necessarily reflect LA function independently [40].

Echocardiography is the first-line imaging modality. The consensus identifies TTE as the initial imaging technique for evaluating suspected atrial cardiomyopathy because it is widely available, non-invasive, and provides comprehensive assessment of atrial size and function. Standard echocardiography measures left atrial diameter and, preferably, LA volume indexed to body surface area, while Doppler techniques and speckle-tracking strain imaging provide information regarding atrial reservoir, conduit, and contractile function.

Echocardiography is particularly valuable for assessing left atrial enlargement, evaluating atrial mechanical function, identifying associated structural heart disease (e.g., valvular disease or ventricular dysfunction), and serial follow-up of atrial remodeling. However, the consensus notes that atrial enlargement is often a late manifestation of ACM, and normal atrial size does not exclude significant microscopic fibrosis or electrical remodeling. From a clinical perspective, TTE should be considered the first-line investigation in patients with suspected ACM because it provides an accessible assessment of atrial morphology and function while simultaneously evaluating concomitant cardiac disease [23].

Cardiac MRI is precise and highly reproducible and has now become the gold standard for evaluating LA volume and function. Late gadolinium enhancement (LGE) imaging is a non-invasive, well-established technique for detecting atrial fibrosis. The imaging agent gadolinium is taken up and released into the bloodstream quickly by healthy myocardial tissue, but it dissipates more slowly from diseased tissue with interstitial fibrosis. This property allows visualization of the amount of contrast agent retained in the myocardium at a delayed time (10–30 min) after contrast injection. Therefore, parametric T1-mapping indices offer high diagnostic accuracy for identifying diffuse interstitial fibrosis [41]. LA remodeling characterized by LGE-MRI shows a significant correlation with atrial volume, AF type, and recurrence risk [42]. Additionally, LGE-MRI also contributes for predicting stroke risk in AF patients; LA fibrosis detected through LGE-MRI was an independent predictor of stroke events and significantly increased the predictive power of the CHA_2_DS_2_-VASc score [43]. Some authors attempted to identify LA remodeling and stratify individuals who would benefit from AF catheter ablation through LGE-MRI. They found that extensive LGE (≥30% LA wall enhancement) predicted a poor response to catheter ablation therapy for AF [44]. In recent years, the application of MRI in catheter ablation procedures for AF has received significant attention. Investigators found that identifying anatomical veno–atrial gaps using LGE-MRI in repeat catheter ablation procedures could shorten ablation duration (161 ± 52 min vs. 195 ± 72 min) and decrease the incidence of recurrent atrial tachycardia (30% vs. 61%) [45]. Some investigators utilized LGE-MRI to assess the baseline level of atrial fibrosis in patients with AF and the level of residual fibrosis outside the scar tissue three months post-ablation. Their findings indicated that the level of residual fibrosis could serve as a predictor for AF recurrence [46]. Moreover, several factors further limit the applicability of LGE-MRI. For instance, the atrial wall is relatively thin compared to the resolution of MRI, making its imaging signal susceptible to interference from adjacent tissues or “volume effect”. Additionally, the post-processing of acquired data remains non-standardized. Current algorithms for quantifying atrial fibrosis typically rely on signal intensity comparisons with the patient’s own normal atrial tissue or atrial blood pool signal-to-noise ratio, which lack uniform comparability among different individuals. The evaluation of cardiac MRI measurements for determining ACM is presented in [47].

The EHRA/HRS/APHRS/SOLAECE Expert Consensus on ACM highlights cardiac MRI as the best non-invasive modality for detailed tissue characterization of the atrial myocardium, particularly through late gadolinium enhancement (LGE) imaging. Compared with echocardiography, MRI offers direct visualization of atrial fibrosis, three-dimensional assessment of atrial anatomy, quantification of scar burden, and identification of regional rather than global remodeling.

The EHRA document suggests that cardiac MRI is particularly useful in patients being considered for catheter ablation, patients in whom the degree of atrial fibrosis may influence prognosis, research settings investigating atrial remodeling, and situations where echocardiography is inconclusive. Nevertheless, the consensus also emphasizes important limitations from which thin atrial walls make imaging technically demanding, image quality varies substantially between centers, acquisition and post-processing are not yet standardized, and thresholds for clinically relevant fibrosis remain incompletely validated [35]. Accordingly, cardiac MRI is not recommended as a routine screening test for every patient with ACM but rather as an advanced imaging modality when tissue characterization is expected to influence management.

## 11. Morphometric Indicators: LA Volume Index, Minimum Volume, Sphericity Index

Morphometric evaluation of ACM has shown that LA elevation is common in non-ischemic dilated ACM and suggests chronic elevation of filling pressure, mitral regurgitation (MR) contribution, and atrial remodeling. Cohort studies have found that LA volume index (LAVI) above normal values is a typical feature of ACM, and progressively higher LAVI values are associated with worse outcomes [47,48].

In a cohort study using MRI, the following values for LAVI were identified: normal—21–52 mL/m^2^, mild increase—52–62, moderate increase—63–73, and marked increase—>73 mL/m^2^, and moderate or marked increase was independently associated with higher mortality. These MRI-defined cutoffs provide practical context for interpreting elevated LAVI in the setting of ACM [47].

In the setting of ACM, the minimum LAVI volume provides additional prognostic information. It has emerged as a more significant marker of adverse outcomes in several recent studies. Studies conducted among patients with dilated ACM with HFpEF have shown that minimal LAVI may better reflect atrial dysfunction and increased filling pressure in ACM of non-ischemic origin [49,50].

Individuals with non-ischemic ACM often exhibit altered LA geometry (increased sphericity). Studies using MRI have shown that a higher LA sphericity index correlated with worse phasic atrial function, greater atrial fibrosis, higher rates of HF and hospitalizations, and worse outcomes, even independent of simple volume measurements. Therefore, assessing LA sphericity index as an additional factor may confirm the diagnosis of ACM in addition to assessing LA volume indices [51,52].

## 12. Functional Indicators: PALS, PACS, Conduction/Reservoir/Pump Functions

The most common functional measure in ACM is peak atrial longitudinal strain (PALS), which is a measure of the atrial reservoir voltage function during ventricular systole that quantifies LA stretch/compliance. The normal range for PALS is around 39%, but in healthy patients, the range for PALS is 32–53%, depending on the method [35]. Another functional measure is peak atrial contractile strain (also known as amplifier pump strain) (PACS), which measures the contraction phase and is caused by atrial contraction immediately after the P wave. The normal range for PACS is around 15–17% [53]. Conduction voltage is often calculated as (PALS–PACS) (or sometimes separately, using voltage synchronization), and reflects the passive component in early diastole [54,55]. In normal conditions, conduction voltage is about 23% according to meta-analyses [55]. PALS and PACS values can vary depending on the imaging modality, provider, age, heart rate, and software [53].

The PALS score is a significant prognostic marker. Lower LA reservoir voltage predicts adverse outcomes in heart failure with reduced ejection fraction (HFrEF) (HF severity, hospitalization, and death) and is often a better marker of increased filling pressure than LA volume alone [56]. PALS and PACS parameters predict arrhythmia risk (lower reservoir voltage and reduced contractile voltage are associated with the occurrence of AF and postoperative AF (preoperative total PALS < 28% predicted postoperative AF after coronary artery bypass graft surgery)) [28]. According to meta-analyses, the PALS index can be used as a prognostic tool in individuals with acute or chronic HFrEF as an additional independent parameter for risk stratification in clinical practice [57]. In case of established ACM (non-ischemic or hypertrophic), a detailed study of the LA voltage (reservoir/conduction/pump) complements prognostic information and helps stratify the risk of AF, stroke, and HF [58].

The reduction in LA strain on speckle-tracking 2D echocardiography in patients with mitral and aortic valve disease is useful in predicting clinical outcomes. In the case of MR, PALS was found to decrease with increasing severity of MR and was an independent predictor of mitral valve surgery and postoperative outcomes [59]. A study by Debonnaire et al. in 121 patients with severe primary MR showed that PALS was the most accurate predictor of LA function among all LA functional measures in identifying patients who underwent mitral valve surgery. Participants with LA systolic strain ≤ 24% had worse survival during follow-up, regardless of symptom status [60]. These results were replicated by L. Yang and co-authors in a similar study involving 104 patients with asymptomatic severe MR [61]. In addition, A. Borg et al. demonstrated that LA systolic strain was an independent predictor of postoperative AF in those who underwent mitral valve surgery for severe MR [62].

In mitral valve stenosis, LA systolic strain is impaired even in asymptomatic patients and can predict the development of AF and cardiovascular complications. R. Ancona et al. found reduced LA systolic strain in 101 patients with rheumatic mitral stenosis, the degree of which correlated with worse cardiovascular outcomes over a 3-year follow-up period, regardless of LA volume, age, and mitral valve area. LA systolic strain was also the most important predictor of new-onset AF over a 4-year follow-up period [63].

K. O’Connor and others have shown that in patients with aortic stenosis (AS), LA strain was significantly correlated with reverse LA remodeling in patients after aortic valve replacement [64,65]. Similar to mitral valve disease, LA strain parameters had predictive value for adverse outcomes in patients with AS. In E. Galli et al.’s study of 128 patients with severe AS, decreased systolic LA strain was associated with increased all-cause mortality, worsening HF, and more frequent hospitalization for cardiac disease [66]. LA systolic strain was also a predictor of postoperative AF in patients with severe AS who underwent surgery [67].

## 13. Electrophysiological Indicators: ECG Markers, Mapping

The most important of the electrophysiological indicators are ECG markers and mapping. ECG data can identify altered P-wave morphology or duration, conduction slowing or delay, atrial block, and mechanical desynchrony [68]. P-wave parameters (PWP) reflect the basic structure, size, and electrical activation of the atria. Changes in these factors manifest as PWP abnormalities that can be easily detected on a standard 12-lead ECG and can be used to make clinical decisions. PWP includes P-wave duration, interatrial block, P-wave end force in lead V1, P-wave axis, P-wave voltage, P-wave area, and P-wave dispersion. PWP can be combined to produce a P-wave index (morphology–voltage–P-wave duration ECG risk score). Population-based cohort studies have shown that abnormal PWP is independently associated with a higher risk of AF, ischemic stroke, sudden cardiac death, and dementia. Studies have shown that PWP, alone or in combination (as a P-wave index), improves the prediction of AF or ischemic stroke [68]. A P-wave duration ≥ 120 ms is the cutoff commonly used to diagnose (partial) atrial block, and biphasic morphology in the lower ECG leads is also required to diagnose advanced atrial block [69].

Mapping parameters can be distinguished from low-voltage areas (LVAs) in bipolar or unipolar electroanatomical mapping. When evaluating voltage thresholds, it has been found that a bipolar voltage of <0.5 mV during sinus rhythm is often used to define LVAs, but sometimes <0.3 mV or stricter limits (<0.1 mV) are used in cases of more advanced fibrosis. Voltage thresholds are often lower in the presence of AF mapping [70,71]. Conduction velocity is also important in evaluating mapping data. According to meta-analyses, conduction velocities of the anterior wall of the LA, lower than 0.70–0.88 m/s, were significantly associated with AF recurrence after ablation (adjusted odds ratio (OR) 3.41, 95% CI 1.55, 7.50, *p* = 0.002) [72]. Table 1 presents the assessment of electrophysiological parameters in the presence of ACM events.

Summarizing, the EHRA consensus considers EAM the most direct in vivo assessment of the electrical substrate of ACM. Low-voltage areas identified during invasive mapping often correspond to regions of fibrosis and structural remodeling. However, because EAM is invasive, it is not intended as a diagnostic test for routine clinical evaluation. Patients most likely to benefit from EAM include patients undergoing catheter ablation for atrial fibrillation; patients with persistent or long-standing persistent AF; patients with recurrent AF after previous ablation; and selected patients in whom substrate-guided ablation strategies are being considered. The consensus notes that EAM may improve understanding of the arrhythmogenic substrate and help individualize ablation strategies, but evidence supporting routine substrate-guided intervention remains limited. Thus, EAM should generally be viewed as a procedural tool rather than a screening modality for ACM [35].

## 14. Atrial Fibrosis Assessment

Fibrosis is a central structural hallmark of ACM and reflects progressive extracellular matrix remodeling, fibroblast activation, and collagen deposition within the atrial myocardium. Current concepts emphasize that fibrosis is heterogeneous in distribution and may involve specific anatomical regions of the atria that are particularly susceptible to mechanical stress, inflammation, and electrical remodeling.

In clinical and experimental studies, fibrosis is most identified in LA, although right atrial involvement may also occur in advanced diseases. Common sites of fibrosis include the posterior LA wall, pulmonary vein (PV) antra, LA roof, interatrial septum, LA appendage, mitral isthmus region, Bachmann bundle, and areas adjacent to the coronary sinus. These regions are exposed to increased wall stress, stretch, turbulent flow, and heterogeneous conduction, making them particularly vulnerable to fibrotic remodeling. In patients with AF, fibrosis frequently clusters around the pulmonary vein ostia and posterior LA wall, whereas diffuse bi-atrial fibrosis may be observed in advanced ACM and atrial failure.

There is currently no universally accepted gold standard for atrial fibrosis quantification in ACM; however, several imaging, electrophysiological, and histopathological approaches are used.

First, LGE-MRI is currently the most widely used non-invasive technique for assessing atrial fibrosis. Fibrosis is quantified as the percentage of fibrotic LA wall tissue relative to total LA myocardial volume. The most used classification system is the Utah staging system: Utah stage I stage—<10%, II stage—10–20%, III stage—20–30%, IV sage—>30%. Higher fibrosis burden is associated with AF persistence, recurrence after ablation, thromboembolic risk, impaired LA strain, and HF progression. Burden limitations include: thin atrial walls, image resolution variability, and lack of standardized acquisition protocols.

Second, EAM identifies low-voltage areas (LVAs) as surrogate markers of fibrosis. Common voltage thresholds are as follows: in the case of sinus rhythm fibrosis, bipolar voltage < 0.5 mV; in the case of severe fibrosis/scar, bipolar voltage < 0.2–0.3 mV. Fibrosis burden may be quantified as: percentage of LA surface area occupied by LVAs, total scar area (cm^2^), or regional distribution. Frequently analyzed regions include: posterior wall, anterior wall, septum, roof, and PV antra. EAM correlates moderately with LGE-MRI and histology.

Third, histological assessment remains the reference standard but is limited clinically because tissue samples are rarely available. Quantification methods include: collagen volume fraction (%), Masson trichrome staining, picrosirius red staining, fibroblast density, and extracellular matrix expansion. Fibrosis may be classified as: interstitial, replacement, patchy, or diffuse.

Fourth, although echocardiography cannot directly visualize fibrosis, several parameters correlate with fibrotic remodeling such as reduced LA reservoir strain, increased LA stiffness index, LA enlargement, and impaired booster function. Reduced PALS is increasingly recognized as an indirect marker of atrial fibrosis and mechanical dysfunction.

Recent ESC/HFA consensus documents emphasize multimodal phenotyping rather than relying on fibrosis alone. Current trends include integrating LGE-MRI, voltage mapping, LA strain imaging, biomarkers (Gal-3, NT-proBNP), and genetic profiling.

The 2025 ESC/HFA consensus also stresses that fibrosis alone does not define ACM, because electrical, mechanical, and structural abnormalities may occur at different stages of disease progression [33].

The extent and localization of fibrosis are clinically important because they correlate with AF initiation and maintenance, stroke risk, response to catheter ablation, progression to atrial failure, HF hospitalization, and mortality. Diffuse fibrosis, particularly involving the posterior LA wall and interatrial conduction pathways, is associated with advanced ACM and poorer clinical outcomes.

## 15. Biomarkers: Galectin-3 and Other Biomarkers (NT-proBNP, HS-cTn)

Blood biomarkers play an important role in predicting the course and outcomes of ACM. Among them, galectin-3 (Gal-3), a β-galactoside-binding lectin encoded by the LGALS3 gene, has emerged as an important marker of inflammation and fibrosis. Gal-3 is involved in cell adhesion, macrophage activation, angiogenesis, apoptosis, tissue repair, fibrogenesis, and ventricular remodeling, and is expressed in multiple intracellular and extracellular compartments [76,77,78,79,80].

The biological role of Gal-3 in atrial remodeling has been established in experimental studies, which have shown that Gal-3 can stimulate cardiac fibroblast proliferation, increase collagen I and III synthesis, activate transforming growth factor-β (TGF-β) signaling, promote macrophage recruitment, accelerate extracellular matrix deposition, and enhance the development of atrial fibrosis. These mechanisms contribute to the structural remodeling that occurs in AF [81].

In assessing the association of Gal-3 with atrial fibrosis, one of the most significant clinical trials compared serum Gal-3 levels with histologically confirmed atrial fibrosis obtained during cardiac surgery. The study found that Gal-3 levels >13.65 ng/mL predicted significant atrial fibrosis. Elevated Gal-3 levels independently predicted fibrosis of atrial appendages, and histological fibrosis remained the strongest predictor of postoperative atrial fibrillation. This study identified one of the first direct associations between circulating Gal-3 levels and tissue-confirmed atrial fibrosis [82].

Numerous observational studies have shown that patients with atrial fibrillation have significantly higher levels of Gal-3 than those in sinus rhythm. Gal-3 levels were found to be lowest in healthy controls, intermediate in paroxysmal AF, and highest in persistent AF. This gradient reflected the progression of atrial remodeling [83].

In evaluating the prediction of AF recurrence after catheter ablation, multiple studies have shown that elevated Gal-3 levels before ablation were associated with increased atrial fibrosis, higher AF recurrence after catheter ablation, and poorer sinus rhythm maintenance. Although Gal-3 alone is not accurate enough to guide clinical decisions, it improves risk stratification when combined with imaging and clinical variables [83].

A systematic review and meta-analysis of 28 studies (10,830 patients) found higher Gal-3 levels in patients with AF compared with controls; significantly higher Gal-3 levels in persistent than paroxysmal AF; approximately 45% higher odds of AF with elevated Gal-3 levels; and higher Gal-3 levels associated with recurrence of AF after treatment. These data support the idea that Gal-3 is a marker of atrial remodeling and not just arrhythmia [84].

Current scientific evidence suggests that Gal-3 reflects the fibrotic component of ACM. Studies have shown that elevated Gal-3 levels correlate with left atrial enlargement, decreased atrial mechanical function, increased atrial fibrosis on cardiac MRI, persistent AF, recurrence of AF after ablation, and progression of atrial remodeling. Thus, Gal-3 is increasingly being considered a biomarker of disease severity, rather than the presence of AF alone [83,85].

Elevated Gal-3 levels have been associated with adverse cardiovascular outcomes in both acute and chronic HF, reflecting progressive myocardial fibrosis and remodeling [86,87]. Gal-3 has also been proposed as a biomarker for early HF detection, risk stratification, and treatment selection, including identifying patients who may benefit from anti-inflammatory therapies or statins [88].

In assessing the significance of NT-proBNP as a biomarker, it is worth noting that NT-proBNP is an inactive cleavage fragment that is released along with biologically active B-type natriuretic peptide (BNP) from ventricular and atrial cardiomyocytes in response to myocardial wall stretch, pressure overload, and increased intracardiac filling pressure [17]. Although NT-proBNP has traditionally been used to diagnose and monitor HF, there is increasing evidence that NT-proBNP also reflects atrial remodeling, increased atrial pressure, and mechanical dysfunction before the development of overt heart failure.

Recent reviews have identified NT-proBNP as one of the most reliable circulating biomarkers associated with ACM. In studies, elevated NT-proBNP levels have been correlated with LA enlargement, impaired LA reservoir and contractile force, increased atrial fibrosis, mechanical atrial dysfunction, development and progression of AF, increased risk of cardioembolic stroke, and adverse cardiovascular outcomes [17]. Several clinical studies have shown a significant inverse relationship between NT-proBNP levels and LA strain measured using point-of-load echocardiography, suggesting that NT-proBNP reflects impaired atrial mechanical function even before there is a noticeable increase in cardiac chamber size [17].

Atrial fibrosis is considered a pathological feature of ACM. Although cardiac MRI-LGE remains the gold standard for detecting fibrosis, NT-proBNP has shown moderate to strong correlation with imaging markers of atrial fibrosis. Recent studies have shown that elevated NT-proBNP predicts advanced atrial remodeling and may serve as a surrogate marker in conjunction with echocardiographic assessment. Rather than directly measuring fibrosis, NT-proBNP reflects increased wall tension resulting from fibrotic stiffening and impaired atrial compliance [17].

Multiple observational studies have shown that NT-proBNP levels progressively increase across all atrial disease groups, both in healthy subjects and in patients with ACM without AF, as well as in patients with paroxysmal and persistent AF. Higher NT-proBNP levels are associated with higher AF burden, larger atrial size, and more advanced remodeling. Elevated baseline NT-proBNP levels also predict the onset of AF in previously asymptomatic subjects and its recurrence after catheter ablation or electrical cardioversion [89,90].

One of the most important recent studies evaluating the effect of NT-proBNP on ACM is a secondary analysis of the EAST-AFNET 4 trial conducted in 2025. The investigators quantified ACM using three readily available markers: LA diameter, PR interval, and NT-proBNP concentration. The investigators found that most patients with newly diagnosed AF already had evidence of ACM. Most patients showed signs of ACM at baseline [69% with at least mildly elevated LA size, 23% with prolonged PR interval (≥200 ms), 56% with NT-proBNP > 365 pg/mL]. Severe ACM, defined as the highest tertile of LA size, PR interval, and NT-proBNP, was associated with higher rates of first primary outcome [HR 7.97 (2.32, 27.37); *p* < 0.001]. Early rhythm control was effective with and without ACM (Pinteraction = 0.160). Increasing severity of ACM was associated with higher rates of recurrent AF, stroke, HF, and cardiovascular death. Importantly, early rhythm control therapy remained effective across all levels of ACM severity, suggesting that elevated NT-proBNP levels or progressive remodeling should not preclude rhythm control strategies [91].

In addition to detecting AF, NT-proBNP has emerged as an important biomarker for thromboembolism caused by ACM. Elevated NT-proBNP levels predict ischemic and cardioembolic stroke independently of documented AF, supporting the hypothesis that ACM itself contributes to thrombogenesis [92]. NT-proBNP has also been incorporated into the validated ABC stroke risk score (age, biomarkers, clinical history), where it improves the prediction of stroke risk compared with traditional clinical scores alone [17,93].

Despite its clinical utility, NT-proBNP is not specific for ACM. NT-proBNP levels are affected by HF, advanced age, renal dysfunction, myocardial ischemia, pulmonary hypertension, valvular heart disease, and obesity (which can lead to decreased circulating NT-proBNP levels). Therefore, NT-proBNP should be interpreted in conjunction with imaging, electrocardiographic markers, and clinical evaluation, rather than as a standalone diagnostic marker [17]. In ACM, progressive fibrosis and impaired atrial elasticity increase LA wall tension, stimulating NT-proBNP secretion. Therefore, elevated NT-proBNP levels indicate not only ventricular dysfunction but also the severity of atrial structural remodeling.

In ACM, persistent atrial dilation, inflammation, oxidative stress, and microvascular ischemia contribute to subtle cardiomyocyte injury and apoptosis. These pathological processes promote the continued release of HS-cTn into circulation even in the absence of overt coronary artery disease. Thus, elevated HS-cTn reflects ongoing myocardial damage associated with atrial remodeling rather than simply acute ischemic necrosis [94].

Recent studies have shown that HS-cTn is primarily a biomarker of active myocardial damage resulting from atrial remodeling. Elevated HS-cTn levels have been associated with several features of ACM, including LA enlargement, impaired LA reservoir and contractile function, atrial fibrosis, increased atrial pressure and wall tension, the development and progression of AF, thromboembolic complications, the development of HF, and cardiovascular mortality. Unlike fibrosis biomarkers such as Gal-3, HS-cTn reflects ongoing cardiomyocyte damage and therefore provides additional information about disease activity rather than chronic structural remodeling [94].

Numerous observational studies have shown that HS-cTn levels are significantly higher in patients with AF than in individuals maintained in sinus rhythm. Troponin levels generally increase with increasing AF burden, being lowest in paroxysmal AF and highest in persistent or permanent AF. Elevated HS-cTn levels independently predict the onset of AF in population-based cohorts and recurrent AF after catheter ablation or electrical cardioversion. These data support the concept that chronic myocardial injury contributes to atrial electrical remodeling and maintenance of AF, rather than being a consequence of the arrhythmia alone [95,96].

Although HS-cTn is not a direct marker of fibrosis, elevated levels correlate with imaging markers of atrial remodeling, including increased LA volume, impaired atrial tension, and extensive fibrosis detected by cardiac MRI. Chronic cardiomyocyte injury promotes fibroblast activation and extracellular matrix deposition, linking myocardial injury to progressive structural remodeling. Therefore, HS-cTn is increasingly considered a biomarker reflecting the transition from reversible atrial injury to irreversible atrial fibrosis, especially when interpreted in conjunction with imaging biomarkers [91].

Elevated HS-cTn levels are consistently associated with adverse cardiovascular outcomes in patients with ACM and AF. Elevated HS-cTn levels predict ischemic stroke, systemic thromboembolism, hospitalization for HF, recurrence of AF after rhythm control interventions, and cardiovascular and all-cause mortality The prognostic value of HS-cTn extends beyond patients with AF, suggesting that chronic myocardial damage contributes to the broader clinical syndrome of ACM.

High-sensitivity troponin has been included in several validated cardiovascular risk prediction models. The ABC (age, biomarkers, clinical history) stroke and bleeding risk scores include HS-cTn in combination with NT-proBNP, recognizing that biomarkers reflecting myocardial injury and wall stress significantly improve prognosis beyond traditional clinical risk factors. Current data suggest that combining HS-cTn with biomarkers reflecting additional pathophysiological pathways, including NT-proBNP (atrial wall stress), Gal-3 (fibrosis), soluble ST2 (fibrosis and inflammation), and growth differentiation factor-15 (cellular stress), allows for better characterization of ACM compared with any other biomarker.

Despite its high prognostic value, HS-cTn is not specific for ACM. Elevated levels can occur in a variety of CVD and systemic disorders, including acute coronary syndromes, chronic HF, myocarditis, hypertensive heart disease, hypertrophic cardiomyopathy, chronic kidney disease, pulmonary embolism, and systemic inflammatory disorders. Accordingly, when evaluating ACM, HS-cTn should be interpreted in a broader clinical context and combined with echocardiography, cardiac MRI, and other circulatory biomarkers. Recent reviews emphasize that HS-cTn is best considered a biomarker of chronic myocardial injury rather than a standalone diagnostic biomarker [94].

Overall, current evidence suggests that Gal-3 and NT-proBNP are promising biomarkers in ACM, reflecting inflammation, fibrosis, remodeling, and calcification processes, while also providing prognostic information regarding HF progression and post-interventional outcomes.

This approach aligns with recent consensus documents, particularly the 2025 Heart Failure Association (HFA)/European Society of Cardiology (ESC) Clinical Consensus Statement on ACM, which emphasizes that most molecular biomarkers have not yet reached the level of evidence required for routine clinical diagnosis.

Although substantial progress has been made in elucidating the molecular mechanisms underlying ACM, it is important to distinguish between biomarkers supported by robust clinical evidence and those that remain primarily investigational. While circulating biomarkers reflecting myocardial stress and fibrosis have demonstrated clinical utility in selected settings, many molecular, proteomic, and transcriptomic markers are currently supported predominantly by experimental studies or early translational research and have not yet been incorporated into routine clinical evaluation or guideline recommendations.

Table 2 provides a summary of the molecular markers in the diagnosis of ACM based on research evidence.

Summarizing, the EHRA consensus reviews multiple circulating biomarkers associated with atrial remodeling, including markers of collagen turnover (e.g., procollagen peptides), fibrosis (e.g., Gal-3), myocardial stretch (BNP, NT-proBNP), inflammation (C-reactive protein), myocardial injury (high-sensitivity troponins), oxidative stress and extracellular matrix remodeling [23,35]. Although many biomarkers correlate with atrial remodeling, AF burden, or stroke risk, the document concludes that none has sufficient diagnostic accuracy or validation to serve as a standalone clinical marker of ACM. Important limitations of biomarkers include lack of specificity for atrial rather than ventricular disease, considerable biological variability, absence of standardized thresholds, and insufficient evidence that biomarker-guided management improves outcomes [23,25]. Therefore, biomarkers should currently be regarded as adjunctive research tools or supportive clinical information rather than routine diagnostic tests for ACM.

## 16. Genetics Aspects: Expression of MYL4, TTN, NPPA, SCN5A, MYBPHL and Other Genes

Most of the evidence linking genetic factors to ACM is based on studies of AF. The genetic contribution to AF has been widely described using a variety of methodological approaches, including family linkage studies, identification of rare genetic variants in individuals with AF, or genome-wide association studies [97,98,99,100]. In addition, a variety of genes affected by mutations have been associated with AF, including genes encoding ion channels, gap junctions, structural proteins, endocrine factors, and transcription factors [101]. In addition, some studies have found that the prevalence of rare variants contributing to AF is quite high, ranging from 10% to 16% in patients with early-onset AF [102]. Furthermore, the presence of rare variants in patients with early-onset AF has been associated with higher mortality.

Evidence directly linking genetic factors to non-arrhythmic manifestations of ACM remains limited. However, recent genome-wide association studies (GWAS) have identified several genetic loci associated with ACM-related phenotypes, including LA enlargement and prolonged P-wave duration [103,104]. In addition, whole-exome sequencing studies in patients with fibrotic ACM without AF have identified multiple rare variants potentially involved in atrial remodeling and fibrosis [105].

Experimental animal models have provided further insight into the genetic mechanisms underlying advanced atrial remodeling. In particular, rare variants affecting the atrial-specific sarcomeric protein myosin light chain 4 (MYL4) have been associated with severe atrial dilatation, fibrosis, and marked conduction abnormalities [106]. Many genetic variants identified in ACM overlap with genes implicated in HF, suggesting a shared molecular substrate and a possible increased long-term risk of HF progression. Furthermore, certain variants, including those involving the natriuretic peptide A (NPPA) gene, have also been associated with increased thromboembolic and stroke risk [107,108]. Overall, current evidence suggests that genetic abnormalities are relatively common in ACM, particularly in younger patients, and may contribute to multiple stages of atrial remodeling, fibrosis, and electrical dysfunction. Increasing data plays an important role for variants affecting sarcomere and structural proteins in the pathogenesis of ACM. Nevertheless, the marked genetic heterogeneity of ACM and its clinical implications require further investigation before routine implementation in clinical practice.

In the left ventricular myocardium (not the valve) of aortic stenosis, MYL4, atrial myosin light chain-1 (ALC-1) gene expression is higher. In studies in patients with AV disease, the amount of ALC-1/MYL4 in LV tissue was significantly higher than in the control group, both when measured and when considering pressure overload. In the preoperative AS group, the amount of ALC-1 was significantly higher (~11.2 ± 9.2% of total ventricular LC-1) compared to ~0.3 ± 0.3% in the control group, and after valve replacement this number decreased to ~4.2 ± 3.3%, and ALC-1 levels correlated with LV systolic pressure (r ≈ 0.45, *p* < 0.01) and with wall thickness/pressure and strain indices between groups. The mean ALC1 levels of the patient groups correlated directly with LV systolic and diastolic pressures and wall thickness (r = 0.94–0.98) and, exponentially, with peak systolic circumferential wall stress (r = 0.98), but not with muscle mass or any other parameter [109].

Data sets from other studies replicate the overload relationship. An independent study comparing cardiomyopathy/overload states also found higher atrial/fetal LC-1 (MYL4) fractions in overloaded ventricles, including AS (~43% vs. ~17% in controls), particularly in the subendocardial region [110]. MYL4 is naturally found in atrial tissue and is expressed at low levels in adult ventricular tissue. The appearance of MYL4 in ventricular tissue reflects reactivation of the “fetal/atrial” isoforms in the presence of pressure overload (as in severe AS). In contrast, calcified aortic valve leaflets contain few or no cardiomyocytes, making MYL4 a non-specific marker of aortic valve tissue. Current scRNA sequencing studies of aortic valve leaflets have focused on VIC/VEC and immune cells rather than cardiomyocyte genes [111].

AF is the most common disorder presenting with ACM. AF in the absence of cardiovascular disease or risk factors has traditionally been referred to as “isolated AF”, but many such cases have been shown to have a “genetic basis” [99,112,113,114,115,116]. The first genetic locus for AF was identified “by linkage analysis in a large family sample” when a variant of the KCNQ1 gene was discovered [86]. Subsequent linkage and candidate gene screening studies have confirmed the association between cardiac ion channel variants and “isolated AF”, including additional gain-of-function potassium channel variants, as well as sodium channel and HCN gene variants. Further studies have revealed associations between solitary AF and “rare variants in genes encoding structural protein genes” (e.g., TTN), transcription factors (e.g., TBX5), or endocrine peptides (e.g., NPPA) [117]. While some of these variants affect genes encoding atrial-specific proteins (e.g., NPPA or MYL4), most rare variants identified to date affect genes encoding proteins with both atrial and ventricular expression [100,118,119].

Recent genome sequence analysis data have been provided important additional insights into the association of rare gene variants and solitary AF in large-scale population-based studies [120,121,122]. Therefore, genetic analysis of patients with early-onset AF is very important to clarify genetic information, since about 60% of such patients’ AF is related to the expression of certain genes [123,124]. The studies also highlight sex differences in MYL4 gene expression. To assess MYL4 gene expression, 756 ventricular myocardium samples from 668 individuals were analyzed using a semi-automated cell profiling method in five cardiac tissue microarrays (TMAs). The percentage of MYL4-positive cells was significantly higher in men across all five TMAs, regardless of disease status (*p* = 8.66 × 10^−15^). Higher MYL4 expression was also associated with hypertrophic ACM (*p* = 6.3 × 10^−4^), but no significant associations were found with sudden cardiac death or other ACM variants [125]. It is noteworthy that the prevalence of rare gene variants detected in the population is highly dependent on the screening method used. In a study of 255 patients with early-onset isolated AF in the absence of known clinical risk factors (i.e., <60 years), screening was performed using a pre-specified set of 43 genes previously associated with ventricular cardiomyopathy [111]. In this cohort, 3.1% of patients had rare gene variants, half of which were associated with the TTN gene. In a recent study, 1293 patients with early-onset AF were screened for rare gene variants using a large panel of genes, including 145 genes associated with ACM and channelopathies. Of the total population, 10.1% of patients had rare gene variants, including 16.8% of patients with AF onset before the age of 30 years, and in most cases, the pathogenic genes were associated with ACM [102]. In addition, the prevalence of AF disease-associated gene variants was 9% in patients with preserved LV ejection fraction (>50%). ACM-associated genes were eight times more common than channelopathy variants, with TTN variants being the most common. Other studies have also confirmed the expression of certain genes in early AF cases and associated this with ACM [108,126,127,128,129]. Table 3 presents genetic family linkage studies associated with isolated AF to the identified rare genetic variants.

Taken together, these data suggest that the genetic basis of early-onset “isolated AF” is essential, especially when analyzed in comprehensive screening using modern multigene ACM and arrhythmia panels. Furthermore, rare gene variants affecting non-atrial genes are more common than variants affecting atrial genes. However, the reasons why alterations affecting non-atrial genes sometimes cause isolated AF rather than more “generalized syndromes” such as HF are still poorly understood. A strong polygenetic predisposition to AF in some of these patients may be part of the explanation [128].

Atrial remodeling assessment is most performed in patients with rare gene variants associated with ACM, such as AF. Because AF is a major contributor to atrial remodeling, it is difficult to know how much of the atrial remodeling is due to the underlying rare gene variant, as opposed to AF itself. However, there is some evidence of atrial remodeling in patients with rare gene variants in the absence of AF. Genetic studies have linked rare gene variants, whose expression is specific to the atria, to features of atrial remodeling. For example, one study reported idiopathic atrial dilatation and atrial arrest associated with a variant in the NPPA gene [87]. In this study of 13 patients, electroanatomical mapping demonstrated areas of low voltage in the right atrium (RA), indicating fibrotic atrial changes. Similar results have been observed in patients with a MYL4 variant who developed atrial arrest and atrial systolic dysfunction despite no prior history of AF [106]. Like isolated AF, rare non-atrial-specific gene variants can also manifest as isolated atrial remodeling. In a recent study of 33 patients with primary fibrotic ACM without AF, 21 of 33 patients had rare variants in 19 ACM-associated genes, the most common of which was TTN [105].

Although the association between genetic factors and atrial remodeling is difficult to establish in the presence of AF, some studies have shown particularly significant changes in atrial remodeling in patients with ACM-associated variants and AF. Variants in the MYH7 gene are associated with significant LA fibrosis in paroxysmal AF [131]. Since no ventricular abnormalities were observed at presentation and since paroxysmal AF does not appear to cause ongoing remodeling, selective structural abnormalities in the atrium were likely the cause, rather than the consequence, of paroxysmal AF. Some studies also report significant evidence of atrial remodeling associated with rare variants affecting Na+ channel genes such as SCN5A, including fibrotic processes associated with ACM, ranging from sick sinus syndrome to atrial arrest [98,106,132]. The association between atrial remodeling and rare gene variants in patients with AF is also supported by population-based studies. A cohort study of 94 patients with AF that looked for rare gene variants associated with ACM found a higher frequency of carriers of gene variants in patients with atrial dilatation. Recent studies have confirmed the importance of a broader genetic background in atrial remodeling and provided the first evidence that the haplotype marked by rs2200733 (PITX2 gene) alters the electrical remodeling of the LA and is a factor in the long-term outcome of AF ablation. The molecular mechanisms underlying these changes require further investigation [133,134].

There are also unresolved questions regarding the expression of structural/contractile genes. In rare variants of TBX5 and MYL4, bradyarrhythmia and pacemaker support are particularly common. It would be useful to better understand the basis of this specific feature and whether it could provide insights into the sinus node dysfunction that often manifests in AF as a tachy-brady syndrome. Stroke is a particularly common feature of AF that markedly impairs atrial contractility, including cases caused by disease-causing gene variants in SCN5A and MYL4. These and other rare variants of AF genes can inhibit atrial contraction to the point of atrial arrest. It is still unclear whether the marked change in atrial contractility is due to the primary effect of the genetic variant or to the secondary effect of the associated atrial structural rearrangement. Sinoatrial node (SAN) dysfunction is frequently associated with atrial arrhythmias, particularly tachy-brady syndrome, and represents an important feature of hereditary AF, often leading to pacemaker implantation. The atrial-selective protein MYL4 (myosin light chain-4), which plays a key role in atrial contractility, has been implicated in familial AF and atrial cardiomyopathy (ACM). Pathogenic MYL4 variants are associated with early SAN dysfunction, progressive atrial remodeling, and increased pacemaker requirement. Experimental studies using genetically modified rats carrying the MYL4 p.E11K mutation demonstrated significant impairment of SAN function compared with wild-type controls [135]. Mutant animals showed prolonged sinus node recovery time, reduced SAN conduction velocity, impaired calcium handling, decreased funny current and L-type Ca^2+^ current densities, and increased fibrosis within the SAN region. These findings suggest that MYL4 mutations contribute to progressive SAN dysfunction through combined disturbances in ion-channel activity, calcium homeostasis, and structural remodeling, providing important mechanistic insights into SAN abnormalities in ACM.

The researchers found that a particular genotype of MYL4 was associated with AF recurrence. In the study, 246 AF patients who underwent cryoablation and were followed up for AF recurrence were studied. The researchers found that the C allele and CC genotype of rs4968309 and the A allele of rs1515751 were associated with AF onset both before and after adjustment for variables (sex, age, AH, and diabetes). AF type and LA size differed between the rs4968309 genotypes. In addition, the CC genotype of rs4968309 significantly increased the risk of AF recurrence after cryoablation. The prevalence of AH was associated with rs1515752, and LA size with rs2071438 genotype. The authors concluded that the MYL4 polymorphisms located in the rs4968309 strand, the C allele, and the CC genotype were associated with AF onset and recurrence. In addition, the strand A allele of rs1515751 had a significant association with AF onset. MYL4 polymorphisms may predict AF onset and prognosis after ablation in patients with AF in the absence of structural heart disease [136]. Recent studies have revealed that the MYL4 gene plays a significant role in muscle development and is actively involved in molecular regulatory mechanisms in skeletal muscle. The expression of the MYL4 gene gradually increased with the extension of myogenic differentiation time. Myoblast function assay showed that overexpression of the MYL4 gene inhibited proliferation and promoted apoptosis and differentiation. Inhibition of MYL4 expression showed the opposite result. The results obtained improved researchers’ understanding of the molecular mechanisms of muscle development and provided a solid theoretical basis for further investigation of the role of the MYL4 gene in muscle development [137,138]. Some studies have shown that serum MYL4 levels in patients with AF were decreased (0.6 ± 0.2) compared to controls (0.1 ± 0.6) (*p* < 0.01), and were negatively correlated with transforming growth factor, procollagen type I C-terminal propeptide, and LA diameter (r = −0.2389, *p* < 0.05; r = −0.5174, *p* < 0.01; r = −0.3191, *p* < 0.01). Low MYL4 levels in patients with AF were associated with a worse prognosis. Serum MYL4 levels and AF type were independent risk factors for AF recurrence after radiofrequency ablation [139].

Myosin-binding protein H-like (MyBP-HL) has recently emerged as a novel atrial-enriched sarcomeric protein with an important role in cardiac physiology and disease. Encoding by the MYBPHL gene, MyBP-HL belongs to the myosin-binding protein family and shares structural homology with the C-terminal domains of cardiac myosin-binding protein-C (cMyBP-C). Unlike cMyBP-C, MyBP-HL is predominantly expressed in the atrial myocardium and the ventricular conduction system, suggesting a specialized role in atrial contractile function and electrical conduction. Although its precise biological function and interaction with cMyBP-C remain incompletely understood, growing evidence indicates that MyBP-HL is essential for maintaining normal cardiac function [140].

Recent experimental studies have demonstrated that disruption of the MYBPHL gene in mice results in cardiomyopathy accompanied by conduction abnormalities and arrhythmias, highlighting the critical role of MyBP-HL in preserving myocardial structure and electrophysiological integrity [141]. These findings support the concept that mutations in atrial-enriched genes can produce a primary atrial myopathy, or ACM, which may subsequently contribute to broader cardiovascular dysfunction. Consequently, MYBPHL represents a promising candidate gene for understanding the molecular mechanisms underlying ACM and its associated arrhythmic and structural phenotypes.

Whole-genome sequencing has identified a rare nonsense variant (R255X) in the *MYBPHL* gene, which encodes MyBP-HL, a recently characterized member of the myosin-binding protein family with predominant atrial expression. Unlike its closely related paralog *MYBPH,* which is also located on chromosome 1, *MYBPHL* exhibits markedly higher expression in the left atrium than in the left ventricle, suggesting a specialized role in atrial myocardial structure and function [142].

Functional studies demonstrated that MyBP-HL is a myofilament-associated protein localized within atrial cardiomyocytes, whereas the truncated protein produced by the R255X variant fails to incorporate into the sarcomeric myofilament. Human cellular models further showed reduced expression of the mutant *MYBPHL* allele, indicating that the pathogenic variant likely results in loss of protein function through impaired expression and defective sarcomeric integration [142].

The physiological consequences of *MYBPHL* deficiency have been investigated in genetically modified mouse models. Echocardiographic assessments of both heterozygous and homozygous *MYBPHL* knockout mice at 12 weeks of age demonstrated moderate cardiac dysfunction characterized by increased left ventricular internal diameter during diastole, reduced fractional shortening, and significant atrial enlargement, as reflected by an increased atrial-to-heart weight ratio. These findings indicate impaired systolic performance accompanied by ventricular remodeling and atrial hypertrophy, despite the predominantly atrial expression of MyBP-HL [142].

More recent investigations have extended these observations by demonstrating that disruption of *MYBPHL* produces not only ventricular dysfunction but also abnormalities of the cardiac conduction system and increased susceptibility to arrhythmias, emphasizing the essential role of MyBP-HL in maintaining both myocardial contractility and electrical stability [141]. The combination of ventricular dilation reduced systolic function, conduction defects, and atrial remodeling supports the concept that *MYBPHL* deficiency contributes to a complex cardiomyopathic phenotype involving both structural and electrophysiological abnormalities.

From a clinical perspective, the *MYBPHL* R255X variant appears to be an uncommon but potentially pathogenic genetic alteration. Among 198 individuals with cardiomyopathy who underwent whole-genome sequencing, the R255X variant was identified in one patient. Although rare in the general population, its observed frequency exceeded that expected based on population prevalence and the marked genetic heterogeneity of dilated cardiomyopathy, suggesting that loss-of-function variants in *MYBPHL* may represent an underrecognized genetic contributor to inherited cardiomyopathy and arrhythmogenesis [141,142]. Collectively, these findings establish *MYBPHL* as a novel atrial-enriched sarcomeric gene whose disruption links atrial dysfunction with ventricular remodeling and conduction abnormalities, supporting its emerging role in the molecular pathogenesis of ACM.

Summarizing the findings of the analyzed studies, it can be stated that the assessment of genetic polymorphisms and genotypes in ACM with aortic and mitral valve pathology is a significant factor that can show that the expression of certain genes (MYL4/ALC-1 and TTN) may be associated with disorders encoding certain structural-sarcomeric proteins in the atria, which lead to atrial remodeling phenomena and changes in atrial function and structure, which may subsequently predispose to the manifestation of clinical signs such as AF or HF and may have an even greater risk of developing early health disorders or the risk of early death. The researchers found that the C allele and CC genotype of rs4968309 and the A allele of rs1515751 were associated with early onset of AF, while the prevalence of AH was associated with rs1515752, and the size of the LA was associated with the genotypes of rs2071438. Additional studies documenting the enrichment of *MYBPHL* truncations in dilated cardiomyopathy populations are needed to develop statistical evidence for *MYBPHL* as an ACM gene.

Summarizing all the findings from diagnostics implications, the presentation of ECG, imaging, biomarkers changes, and genetics domains collectively contribute to the multidomain characterization of ACM.

## 17. Clinical and Prognostic Significance of ACM

In terms of the clinical and prognostic significance of ACM, it can be stated that this pathology is associated with a significantly increased risk of AF, and in its presence, there is a significantly increased risk of ischemic stroke, which in turn worsens the risk of AF due to disturbances in the atrial fibrosis/remodeling cycle [143]. Multiple cohort studies and meta-analyses show that markers of ACM, such as elevated NT-proBNP, abnormal P-wave end force in lead V1, increased LA, and fibrosis, are associated with a higher risk of ischemic stroke, regardless of clinically recognized AF. This supports the concept that the atrium itself may be a source of thromboembolism even before AF is detected [144]. Previous studies have shown that worse outcomes after stroke in ACM are associated with greater initial stroke severity and worse prognosis [145]. Reduced success of heart rate control therapy in the presence of established atrial fibrosis on MRI predicts significantly higher recurrence of AF after catheter ablation and is associated with worse procedural outcomes [146]. Studies have found that HF, cognitive decline, and mortality link biomarkers of ACM with HF progression, dementia, and higher mortality, although the causal and pathogenic pathways are still being elucidated [147].

No single study defines the diagnosis of ACM, as the pathology is multimodal. When evaluating electrocardiographic data, P-wave end force abnormalities in lead V1 have been most assessed in cohort studies [148]. When evaluating blood biomarkers such as NT-proBNP, their significance is more associated with repeated random stroke, AF determination after stroke, and overall atrial dysfunction. Several meta-analyses have shown that NT-proBNP is a useful marker for selecting individuals for long-term rhythm monitoring after cryptogenic stroke [144,149]. When evaluating echocardiographic data, LA diameter, LAVI value, LA voltage, and reservoir/amplifier function measurements are prognostic indicators determining the outcome of ACM and the risk of arrhythmia [150,151].

In assessing the value of cardiac MRI, LGE-MRI quantifies LA fibrosis, predicts the degree to which it anticipates AF recurrence after ablation (DECAAF study), and is associated with a higher risk of embolism. MRI provides direct information about the structural substrate of the LA but is not universally available or standardized [152,153,154].

This raises a broader conceptual issue in ACM research: atrial disease is multidimensional, involving fibrosis, inflammation, adipose infiltration, microvascular dysfunction, myocyte degeneration, autonomic remodeling, and electromechanical dissociation. LGE-MRI captures only part of this spectrum. Therefore, relying on a single imaging biomarker to define ACM risks oversimplifying a biologically heterogeneous condition. Another important concern is temporal variability. Fibrosis is often treated as a static substrate, yet atrial remodeling evolves dynamically with AF burden, heart failure, obesity, AH, and systemic inflammation. A single LGE snapshot may therefore inadequately represent the ongoing pathophysiology of ACM. There is also a translational gap between imaging and electrophysiology. Low-voltage regions do not consistently overlap with LGE regions, and electrically abnormal tissue may exist without visible enhancement. Conversely, enhanced regions may not always be arrhythmogenic [155]. This weakens the rationale for directly targeting all MRI-defined abnormalities during ablation.

Future progress will likely require higher spatial resolution imaging, standardized acquisition and post-processing protocols, integration of multimodal biomarkers (MRI, voltage mapping, strain imaging, biomarkers, AI phenotyping), and validation against histopathology and clinical outcomes rather than imaging appearance alone [156].

In summary, LGE-MRI remains a highly promising tool for studying atrial cardiomyopathy, but current fibrosis quantification methods are still limited by resolution constraints, methodological heterogeneity, uncertain biological specificity, and inconsistent reproducibility. These challenges should temper overly deterministic interpretations of “fibrosis burden” in both clinical trials and individualized AF management.

Importantly, enhancement thresholds were determined by “expert inspection,” introducing operator dependency into the methodology [157]. This raises concerns about reproducibility, especially across multicenter studies where scanner hardware, field strength, acquisition parameters, and technical expertise vary considerably. Indeed, one of the underappreciated findings from DECAAF was that 17.3% of MRI studies were nondiagnostic because of poor image quality. The causes included: incorrect recovery timing, respiratory motion, arrhythmia-related gating problems, high heart rate, obesity, incomplete atrial coverage, and hardware limitations [157]. This finding is clinically important because patients with persistent AF—the group most likely to exhibit advanced atrial cardiomyopathy—are also those most difficult to image reliably due to irregular RR intervals and motion artifact. Thus, technical limitations disproportionately affect the exact population in whom fibrosis quantification is considered most clinically relevant.

Despite confirming that higher fibrosis burden predicts worse outcomes, MRI-guided fibrosis ablation did not significantly reduce arrhythmia recurrence compared with pulmonary vein isolation alone [158]. This finding challenged the assumption that all MRI-defined fibrotic tissue represents an appropriate ablation target. The DECAAF II investigators themselves acknowledged several explanations: not all fibrosis is arrhythmogenic, fibrosis texture and spatial distribution may matter more than total burden, and current imaging cannot distinguish mechanistically important substrates from bystander scar [146]. This distinction is crucial for understanding atrial cardiomyopathy. Another unresolved issue concerns spatial heterogeneity. Subsequent DECAAF II analyses demonstrated that fibrosis distribution may carry prognostic importance independent of total fibrosis burden. Regional fibrosis near the left atrial appendage or pulmonary veins may confer different arrhythmogenic properties than diffuse fibrosis elsewhere [159]. Thus, reducing ACM to a single percentage value may fail to capture clinically meaningful substrate complexity.

From a clinical perspective, the DECAAF experience suggests several important conclusions. First, MRI-defined atrial fibrosis should be interpreted as an imperfect imaging biomarker rather than a direct histological measurement. Second, fibrosis staging has prognostic value but limited precision for individualized substrate targeting. Third, current LGE methodologies remain vulnerable to acquisition variability, operator dependency, and limited reproducibility. Fourth, ACM cannot be fully characterized by fibrosis imaging alone. Fifth, multimodal phenotyping integrating MRI, electroanatomic mapping, atrial strain imaging, circulating biomarkers, and clinical phenotype will likely be required for accurate characterization of atrial disease.

In ambulatory rhythm monitoring studies, atrial extrasystoles and more frequent episodes of atrial paroxysmal tachycardia indicate atrial electrical instability and a higher risk of AF and stroke [160]. It is very important to rely on the EHRA consensus documents when identifying ACM events, which recommend combining existing tests (ECG, biomarker, and imaging studies) to identify atrial contractility disorders and timely referring these individuals to a personal health care facility with appropriate specialists [1].

Timely identification and control of AF risk factors are essential for assessing the outcome of treatment and disease management in AF. Control of AH, obesity, sleep apnea, diabetes, and valvular disease remains essential preoperatively, as these factors slow the progression of atrial remodeling and reduce the burden of complications associated with AF. The consensus documents adopted by the EHRA emphasize aggressive management of the above risk factors [23]. The use of some anticoagulation therapy in ACM without documented AF is also debatable.

The ARCADIA randomized trial, which compared the use of direct anticoagulants and aspirin in patients with recent cryptogenic stroke and ACM without AF, did not demonstrate a benefit of anticoagulation in preventing recurrent stroke. This neutral result was an important finding, as ACM was associated with AF risk, but it did not prove that conventional anticoagulation in ACM without AF reduced the risk of recurrent stroke. The authors of the study recommended paying more attention to heart rate monitoring and individualized care rather than conventional anticoagulation [161]. The ARCADIA trial was one of the most important mechanistic stroke trials of the last decade because it directly tested the hypothesis that ACM—even in the absence of overt AF—could represent a thromboembolic substrate warranting anticoagulation. The trial randomized patients with embolic stroke of undetermined source (ESUS) and evidence of atrial cardiopathy to apixaban versus aspirin yet showed no reduction in recurrent stroke with anticoagulation [161].

This neutral result has major implications for the future conceptualization of ACM. Rather than disproving atrial cardiopathy, ARCADIA likely exposed several unresolved problems involving biomarker selection and thresholding, mechanistic heterogeneity of ESUS, biological specificity of ACM markers, and incomplete understanding of atrial thrombogenesis.

A central issue is that ARCADIA operationalized atrial cardiopathy using relatively simple surrogate biomarkers: P-wave terminal force in V1 (PTFV1) > 5000 μV·ms, NT-proBNP > 250 pg/mL, or left atrial diameter index (LADI) ≥ 3 cm/m^2^ [146]. These thresholds were derived from observational associations with stroke risk, but observational correlation does not necessarily identify a causal cardioembolic mechanism. This distinction is critical. The first major criticism is that the thresholds may have been biologically too non-specific and insufficiently severe to identify a truly thrombogenic atrial substrate. For example, NT-proBNP > 250 pg/mL is only modestly elevated and may reflect aging, AH, diastolic dysfunction, renal impairment, HF tendency, or generalized cardiovascular stress, rather than a distinct atrial thromboembolic phenotype. Similarly, PTFV1 is influenced by multiple extracardiac and conduction-related factors and has imperfect reproducibility. LA diameter is also a crude geometric parameter that incompletely captures atrial remodeling compared with modern volumetric or strain-based assessment. Importantly, post hoc ARCADIA analyses demonstrated that the selected biomarkers were only moderately predictive of future AF. NT-proBNP and LADI retained stronger associations, whereas PTFV1 performed weekly. Even combined biomarker discrimination showed only modest calibration [161]. This suggests that many enrolled patients may not have had advanced ACM capable of producing atrial thrombosis. In other words, ARCADIA may have diluted a truly high-risk subgroup within a broader, biologically heterogeneous ESUS population.

A second major issue concerns the underlying stroke mechanism itself. ARCADIA implicitly assumed that atrial cardiopathy primarily causes stroke through thromboembolism analogous to AF. However, ESUS is mechanistically heterogeneous and includes occult large-artery atherosclerosis, non-stenotic plaque embolism, ventricular disease, cancer-associated thrombosis, paradoxical embolism, small-vessel disease masquerading as embolic stroke, and systemic endothelial dysfunction. Thus, even when atrial cardiopathy biomarkers are present, the actual recurrent stroke mechanism may not be atrial thromboembolism. Anticoagulation would therefore fail to demonstrate benefit because the treated mechanism is not dominant. This is consistent with broader ESUS experience. Trials such as NAVIGATE ESUS and RE-SPECT ESUS also failed to show superiority of anticoagulation over aspirin, suggesting that ESUS is not a uniformly cardioembolic entity. A third important implication is that atrial cardiomyopathy may not be binary. ARCADIA used dichotomous thresholds (“present” vs. “absent”), yet ACM is likely a continuous and multidimensional biological process involving fibrosis, atrial myopathy, inflammation, endothelial dysfunction, impaired atrial mechanics, electromechanical dissociation, and prothrombotic remodeling.

Simple threshold-based biomarker definitions may therefore inadequately capture the true severity or thrombogenicity of atrial disease. This may explain why stronger biomarkers appear more promising than those used in ARCADIA. Several emerging markers likely better reflect advanced ACM: left atrial reservoir strain, atrial fibrosis on LGE-MRI, left atrial appendage dysfunction, prolonged atrial electromechanical delay, high-density electroanatomic abnormalities, and combined multimodal risk scores. The failure of ARCADIA therefore likely reflects insufficient phenotypic precision rather than absence of an atrial mechanism altogether. Another underappreciated issue is temporal mismatch. Atrial thrombogenesis may occur intermittently and dynamically. Patients could have transient periods of atrial dysfunction or subclinical AF not captured during trial enrollment. In ARCADIA, AF surveillance was relatively pragmatic rather than based on systematic prolonged implantable monitoring for all participants. As a result, some patients may have developed clinically meaningful AF later, while others never had a truly emboli genic atrial substrate [148]. Interestingly, subsequent ARCADIA analyses showed that biomarker severity correlated with future AF detection, particularly NT-proBNP. This supports the broader concept that ACM and AF exist along a disease continuum. However, ARCADIA also suggests that not all forms of ACM are sufficiently thrombogenic to justify empiric anticoagulation [148].

This has profound implications for future ACM management. First, future trials will likely require more biologically specific phenotyping rather than simple threshold-based screening. The field is moving toward: multimodal ACM characterization, quantitative atrial imaging, strain-based functional assessment, fibrosis quantification, AI-derived ECG phenotyping, and integrated biomarker panels. Second, future anticoagulation strategies may need enrichment for patients with more advanced or mechanistically coherent atrial disease. For example, severe atrial dysfunction, extensive fibrosis, spontaneous echo contrast, left atrial appendage impairment, or recurrent atrial ectopy may identify patients more likely to benefit from anticoagulation than isolated mild biomarker abnormalities. Third, ARCADIA shifts the field away from the simplistic idea that “atrial cardiopathy equals anticoagulation indication”. Instead, ACM likely represents a spectrum in which some patients primarily have arrhythmic risk, some have thromboembolic risk, and others have generalized vascular dysfunction without a dominant cardioembolic mechanism.

Finally, ARCADIA reinforces that AF itself may not be the sole driver of embolic stroke. Rather, AF may function as a marker of advanced atrial disease. Yet the converse is also important: not all atrial disease reaches the threshold necessary to generate clinically important thrombosis. Thus, the central lesson of ARCADIA is not that ACM is irrelevant, but that current biomarker definitions remain insufficiently precise to identify the subgroup in whom atrial disease is the dominant and anticoagulation-responsive stroke mechanism.

Studies have also assessed the success of interventional trials. In terms of ablation and heart rate control, the degree of atrial fibrosis was found to be a determinant of ablation success, and patients with more extensive atrial fibrosis had a higher incidence of disease recurrence after ablation. Some studies of MRI-guided fibrosis ablation have failed to demonstrate a clear advantage over fibrosis-guided strategies. Assessment of fibrosis/ACM should be considered in clinical decision-making, but the evidence for changing the standard ablation strategy is conflicting [146].

When evaluating the prognostic factors of ACM disease, it was found that individual markers may have prognostic relevance, but this does not necessarily imply that ACM as a syndrome is causally involved [162,163].

## 18. Limitations

Despite substantial advances in the understanding of ACM, its diagnosis remains challenging because there is currently no universally accepted definition or diagnostic gold standard. Instead, ACM is identified using a combination of structural, electrical, mechanical, and biochemical abnormalities assessed by multiple complementary modalities. These include electrocardiography (ECG; P-wave indices, PR interval prolongation), circulating biomarkers (e.g., NT-proBNP, high-sensitivity cardiac troponin), echocardiography (LA size and functional parameters, including LA strain), cardiac MRI for fibrosis assessment, EAM, and ambulatory rhythm monitoring to detect atrial ectopy or atrial high-rate episodes. However, the diagnostic thresholds, definitions, and interpretation of these modalities vary considerably across studies and clinical practice, resulting in a fragmented approach to diagnosis.

The EHRA expert consensus first emphasized in 2016 that ACM should be regarded as a “conceptual pathological framework rather than a disease entity with universally accepted diagnostic criteria”, reflecting the absence of standardized definitions and validated diagnostic thresholds. More recently, the 2024 EHRA consensus proposed a staging system intended to classify the severity of atrial disease across its clinical continuum. Nevertheless, the document explicitly acknowledges that the proposed criteria require prospective validation, assessment of reproducibility, and correlation with clinical outcomes before widespread implementation in routine practice.

Cardiac imaging represents the cornerstone of ACM assessment, yet important limitations remain. Echocardiography and cardiac MRI routinely evaluate LA size, function, and remodeling, while EAM provides information on atrial voltage abnormalities. However, no guideline-endorsed cutoff values currently exist for LA strain, atrial enlargement, or voltage mapping that specifically define ACM.

LGE-MRI has emerged as the principal non-invasive technique for assessing atrial fibrosis. The landmark DECAAF study demonstrated that greater atrial LGE burden predicts AF recurrence following catheter ablation, supporting the clinical importance of atrial fibrosis. Nevertheless, quantification of atrial fibrosis remains technically challenging and lacks standardization.

A fundamental limitation is that LGE does not directly measure fibrosis but rather visualizes alterations in gadolinium distribution within the extracellular space. Consequently, enhancement represents an indirect imaging surrogate rather than a direct histopathological measurement of collagen deposition. Enhanced regions may reflect a variety of biological processes, including fibrosis, inflammation, edema, adipose infiltration, microvascular injury, prior ablation scar, or non-specific extracellular matrix expansion. Therefore, equating LGE with fibrosis may oversimplify the complex biological substrate underlying atrial remodeling.

Several technical factors further complicate LGE-MRI assessment. The atrial wall is typically only 1–2 mm thick, approaching the spatial resolution limits of clinical MRI. This results in pronounced partial-volume effects, whereby atrial myocardium, blood pool, epicardial fat, and adjacent structures may occupy the same imaging voxel, reducing measurement accuracy. In addition, image acquisition is highly susceptible to respiratory motion, cardiac motion, rhythm irregularity, contrast timing, heart rate variability, and scanner-specific acquisition protocols. Image quality is particularly challenging in patients with persistent AF because beat-to-beat variability compromises ECG-gated acquisition.

Another important limitation is the absence of standardized post-processing methods. Current fibrosis quantification relies heavily on manual or semi-automated segmentation of the atrial wall, and small differences in contour delineation may substantially alter fibrosis estimates because of the thin atrial myocardium. Furthermore, different institutions employ varying thresholding methods, normalization strategies, image intensity ratio techniques, and post-processing algorithms, leading to considerable inter-center variability and limiting reproducibility. Consequently, fibrosis burdens and prognostic thresholds derived from one imaging platform may not be directly transferable to another.

Histopathological validation also remains incomplete. Although several studies have demonstrated correlations between LGE, low-voltage regions, and histological fibrosis, these associations are modest and heterogeneous compared with ventricular cardiomyopathies. Current imaging techniques cannot reliably distinguish electrically active arrhythmogenic fibrosis from structurally abnormal but electrophysiologically irrelevant tissue.

These methodological limitations have important clinical implications. The DECAAF II trial challenged the assumption that MRI-guided ablation of fibrotic atrial substrate provides superior clinical outcomes compared with pulmonary vein isolation alone. Rather than disproving the biological importance of atrial fibrosis, these findings suggest that currently available LGE-MRI techniques may not yet provide sufficiently accurate or mechanistically specific substrate characterization to guide individualized ablation strategies.

Ambulatory ECG monitoring provides complementary information regarding atrial electrical remodeling by quantifying premature atrial complexes, atrial tachyarrhythmias, and atrial high-rate episodes. These parameters have consistently been associated with an increased risk of AF, ischemic stroke, and cardiovascular events. However, no consensus currently exists regarding the burden, frequency, or duration of atrial ectopy or atrial high-rate episodes required to define clinically significant ACM, limiting their incorporation into standardized diagnostic algorithms.

The absence of standardized diagnostic criteria has significant implications for both clinical practice and research. Existing studies use heterogeneous combinations of ECG findings, biomarkers, imaging parameters, electrophysiological measurements, and composite scoring systems, making comparisons across studies difficult and limiting the ability to perform robust meta-analyses. Furthermore, although trials such as ARCADIA operationalized ACM using predefined criteria—including abnormal ECG findings, LA enlargement, and elevated NT-proBNP concentrations, these definitions have not been universally adopted and remain primarily research tools rather than routine clinical diagnostic standards.

Despite considerable progress, several important challenges remain unresolved. Universal diagnostic thresholds have yet to be established for LA strain, fibrosis burden, EAM, or circulating biomarkers. The recently proposed EHRA staging system represents an important conceptual advance but requires prospective validation, demonstration of reproducibility across imaging platforms, and confirmation that individual stages correlate with meaningful clinical outcomes.

At present, no molecular biomarker demonstrates sufficient sensitivity, specificity, or validation to serve as a standalone diagnostic marker for ACM. Biomarkers such as NT-proBNP, HS-cTn, Gal-3, and soluble ST2 may provide complementary information regarding atrial stress, fibrosis, and myocardial injury, but their interpretation should always be integrated with clinical assessment, electrocardiographic findings, and multimodality cardiac imaging. In contrast, emerging molecular biomarkers—including microRNAs, extracellular vesicles, proteomic and metabolomic signatures and genetic profiling—have substantially advanced the understanding of ACM pathophysiology but currently remain investigational. Large prospective multicenter studies, standardized analytical methodologies, and external validation are required before these biomarkers can be incorporated into routine clinical practice.

Equally important is the unresolved question regarding the causal relationship between ACM and AF. Increasing evidence supports a bidirectional interaction in which atrial remodeling promotes the development and maintenance of AF, while AF itself accelerates progressive structural, electrical, and functional remodeling of the atria. Defining the temporal sequence of these processes remains one of the major challenges in contemporary atrial research.

## 19. Conclusions and Future Research Directions

When assessing the challenges of implementing standardized testing methods, it should be noted that without standardized diagnostics, most ACM diagnostic situations remain primarily research tools rather than practical clinical diagnostic methods. In summary, it can be stated that there is no standardized diagnostic test method for ACM to date. Current methods rely on heterogeneous markers (ECG, biomarkers, imaging, monitoring), and the limits of the tests vary. This lack of standardization hinders both the comparison of tests and their application in clinical practice.

Although significant progress is being made in understanding ACM, several important challenges still limit its routine clinical use. These include the absence of universally accepted diagnostic criteria, insufficiently standardized staging systems, uncertainty regarding anticoagulation strategies in patients without AF, a lack of dedicated randomized clinical trials, and difficulties in determining whether atrial remodeling is reversible or permanent. Addressing these gaps remains a major focus of ongoing research.

As for major unresolved challenges, several barriers remain before these ideas become routine practice, such as the absence of a universally accepted diagnostic criteria, lack of standardized staging systems, uncertain thresholds for anticoagulation without AF, limited randomized trials focused specifically on ACM, and the difficulty in distinguishing reversible from irreversible remodeling. These are currently major research priorities.

Looking ahead, management of ACM is expected to move toward earlier diagnosis, more precise patient characterization, prevention-oriented strategies, therapies aimed at modifying the underlying disease process, and a more integrated approach to stroke, arrhythmia, and heart failure care. Ultimately, ACM may come to be recognized and treated as an independent cardiovascular disease rather than merely a precursor to AF.

The future management of ACM is likely to evolve toward earlier detection, precision phenotyping, prevention-focused care, disease-modifying therapy, and integrated stroke–arrhythmia–heart failure management. The broader implication is that atrial disease may eventually be treated as a primary cardiovascular disorder, rather than simply a precursor to AF.

Future progress will likely depend on integrating multimodal imaging, electrocardiographic assessment, circulating biomarkers, electrophysiological mapping, and emerging molecular and genetic markers into standardized diagnostic algorithms. Such an integrated precision-medicine approach may improve the early identification of ACM, facilitate individualized risk stratification, and ultimately guide targeted preventive and therapeutic interventions before irreversible atrial remodeling and clinical complications develop.

## Figures and Tables

**Table 1:** Evaluation of electrophysiological parameters in the presence of atrial cardiomyopathy events.

Authors	Parameters	Variables	Association with ACM
Fujimoto Y et al., 2018 [73]	P-wave duration and dispersion	Long atrial activation time; conduction heterogeneity.	In patients with chronic AF, after electrical cardioversion, P-wave duration > 120 ms and increased dispersion predicted AF recurrence.
Ciuffo L et al., 2020 [69]	Advanced atrial septal block (ASB)	P-wave duration ≥ 120 ms and biphasic (+/−) morphology in the lower leads (II, III, aVF) reflect conduction delay through the Bachmann bundle, etc.	Patients (*n* = 152) with advanced ASB had higher LAVI min., more fibrosis, and lower LA strain indices.
Schreiber T et al., 2019 [74]	P-wave amplitude	Lower amplitudes in some leads may reflect loss of viable myocardium or fibrotic changes.	Subjects (*n* = 73) with AF: P-wave amplitude in leads I, II, aVR, aVF, V1, V4–V6 showed an inverse correlation with the LVA of the LA (measured on a high-density LA map). In I derivation of P amplitude, <0.062 mV, predicted LVA > 35%.
Weinsaft JW et al., 2014 [75]	P-wave area (in lead V1)	Combines amplitude and duration; reflects overall atrial mass/shape and remodeling.	In a cohort of patients with CHD, P-wave area in lead V1 correlated with LA area (MRI); increased P-wave area was also associated with worse MR and higher pulmonary artery pressure.
Chen LY et al., 2022 [68]	P-wave terminal force (PWTF) in lead V1, morphology, etc.	Long used as markers of LA elevation or abnormality (results are debatable, especially when fibrosis is present, which reduces P-wave amplitude)	PWTF sensitivity/specificity in lead V1 is quite low

**Table 2:** A summary of the molecular markers in the diagnosis of ACM based on research evidence.

Well-Established Clinical Evidence	Emerging Translational Evidence	Experimental Molecular and Genetic Approaches
Galectin-3: One of the best-studied circulating fibrosis biomarkers	Transforming growth factor-β (TGF-β)	Whole-genome sequencing
Galectin-3: Multiple clinical studies show associations with atrial fibrosis, AF recurrence after catheter ablation, and structural remodeling	Connective tissue growth factor (CTGF)	Transcriptomics
Galectin-3: Lacks specificity because it reflects systemic fibrosis and inflammation	Matrix metalloproteinases (MMP-2, MMP-9)	Single-cell RNA sequencing
NT-proBNP and BNP: Reflect atrial wall stress and chamber stretch	Tissue inhibitors of metalloproteinases (TIMPs)	Spatial transcriptomics
NT-proBNP and BNP: Well validated in heart failure and atrial fibrillation	Collagen synthesis markers (PIIINP, PINP, PICP)	Epigenetic profiling
NT-proBNP and BNP: Associated with atrial remodeling and stroke risk	Inflammatory cytokines (IL-6, TNF-α)	Multi-omics integration
NT-proBNP and BNP: Recommended in clinical guidelines for heart failure, but not as standalone ACM biomarkers	Oxidative stress markers	Artificial intelligence-derived molecular signatures
HS-cTn: Marker of ongoing myocardial injury	Circulating extracellular vesicles	
HS-cTn: Predicts AF incidence, stroke, and adverse cardiovascular outcomes	microRNAs (miR-21, miR-29, miR-133, miR-328, miR-425)	
	These biomarkers have demonstrated mechanistic relevance in animal models and small human cohorts but lack standardized assays, validated diagnostic thresholds, and prospective multicenter validation.	These approaches are discussed primarily as contributors to disease susceptibility or mechanistic pathways rather than as clinically applicable diagnostic markers.

**Table 3:** Family linkage studies associated with isolated atrial fibrillation identified rare genetic variants (contractile protein mutation).

Studies	Genes/ Mutation/ Protein	Gene, Characterized by Atrial Expression	Country	Family Links	AF Manifestation	LA Increase	Extracardiac Phenotype
Orr N et al., 2016 [118]	*MYL4*/ c.31G>A/ ALC-1	Yes	Canada	No	Paroxysmal and persistent AF	Yes	No
Gudbjartsson DF et al., 2017 [130]	*MYL4*/ c.234delC/ ALC-1	Yes	Island	Yes	Paroxysmal and persistent AF	Yes	No
Ahlberg G et al., 2018 [126]	*TTN*/ c.107635C>T/ titin	Yes	Denmark	Yes	Paroxysmal and persistent AF	Yes	No

AF—atrial fibrillation, LA—left atrial.

## Data Availability

The data supporting the findings of this study are available from the corresponding author upon reasonable request.

## Abbreviations

ACM	atrial cardiomyopathy
CVD	cardiovascular diseases
LA	left atrium
AF	atrial fibrillation
MRI	magnetic resonance imaging
EHRA	European Heart Rhythm Association
HF	heart failure
RAAS	renin–angiotensin–aldosterone system
TGF-β	transforming growth factor-β
ECM	extracellular matrix
CTGF	connective tissue growth factor
α-SMA	α-smooth muscle actin
TIMP	tissue inhibitors of metalloproteinases
ROCK	Rho-associated protein kinase
GTP	guanosine triphosphate
ROS	reactive oxygen species
EAT	epicardial adipose tissue
DNA	deoxyribonucleic acid
ATP	adenosine triphosphate
miRNA	microribonucleic acid
NF-κB	nuclear factor kappa-light-chain-enhancer of activated B cells
NLRP3	NLR family pyrin domain containing 3
PGC-1α	peroxisome proliferator-activated receptor gamma coactivator 1-alpha
JAK/STAT	Janus kinase
MAPK	mitogen-activated protein kinase
HFA	Heart Failure Association
ESC	European Society of Cardiology
HRS	Heart Rhythm Society
APHRS	Asia Pacific Heart Rhythm Society
SOLAECE	Latin American Society of Cardiac Pacing and Electrophysiology
TTE	transthoracic echocardiography
AH	arterial hypertension
EF	ejection fraction
NT-proBNP	N-terminal pro b-type natriuretic peptide
LGE-MRI	late gadolinium enhancement MRI
HFpEF	heart failure with preserved ejection fraction
LAVI	left atrium volume index
LAVmin.	left atrium volume min.
LAVImin.	left atrium volume index min.
PALS	peak atrial longitudinal strain
LVDD	left ventricular diastolic dysfunction
LV	left ventricular
ECG	electrocardiography
CT	computed tomography
PET-CT	positron emission tomography of the heart-computed tomography
MR	mitral regurgitation
PACS	peak atrial contractile strain
HFrEF	heart failure with reduced ejection fraction
AS	aortic stenosis
PWP	P-wave parameters
LVA	low-voltage areas
OR	odds ratio
ASB	atrial septal block
CHD	coronary heart disease
PWTF	P-wave terminal force
PV	pulmonary vein
EAM	electroanatomical voltage mapping
Gal-3	galectin-3
HS-cTn	high-sensitivity cardiac troponin
AV	aortic valve
TAVR	transcatheter aortic valve replacement
VIC	valvular interstitial cells
HR	hazard ratio
MYL4	myosin light chain 4
NPPA	natriuretic peptide A
ALC-1	atrial myosin light chain-1
MYBPHL	myosin-binding protein-H like
cMyBP-C	myosin-binding protein-C
TMA	tissue microarrays
RA	right atrium
SAN	sinoatrial node
AI	artificial intelligence
ESUS	embolic stroke of undetermined source
LADI	left atrial diameter index
